# Assessing clinical progression measures in Alzheimer's disease trials: A systematic review and meta‐analysis

**DOI:** 10.1002/alz.14314

**Published:** 2024-10-22

**Authors:** Jonathan McLaughlin, William J. Scotton, Natalie S. Ryan, John A. Hardy, Maryam Shoai

**Affiliations:** ^1^ University of Aberdeen NRS Career Researcher Fellow Royal Cornhill Hospital Scotland UK; ^2^ University College London Queen Square Institute of Neurology London UK; ^3^ UK Dementia Research Institute, UCL London UK

**Keywords:** Alzheimer's dementia, amyloid positive, meta‐analysis, outcome measures, progression measures, randomized controlled trials, systematic review

## Abstract

**INTRODUCTION:**

Assessing treatments for Alzheimer's disease (AD) relies on reliable tools for measuring AD progression. In this analysis, we evaluate the sensitivity of clinical progression measures in AD within randomized controlled trials (RCTs) with confirmed positive amyloid (Aβ+) status prior to trial enrollment.

**METHODS:**

Excluding trials targeting non‐cognitive symptoms, we conducted meta‐analyses on progression measures from 25 selected RCTs using R version 4.2.0, along with the *metafor* and *emmeans* libraries.

**RESULTS:**

The Functional Activities Questionnaire (FAQ) demonstrated the greatest sensitivity over 12 weeks. Other cognitive measures demonstrated lower sensitivity. The integrated Alzheimer's Disease Rating Scale (iADRS) and Clinical Dementia Rating‐Sum of Boxes (CDR‐SB) seemed more effective than their individual cognitive components. Neuropsychiatric measures were the least sensitive in measuring progression.

**DISCUSSION:**

Functional measures generally outperformed other measure categories. Purely cognitive domain‐based measures were suboptimal for tracking early AD progression. Ideally, future measures should incorporate both cognitive and functional components to enhance sensitivity.

**Highlights:**

- Concerns remain regarding the limitations of current outcome measures used in AD clinical trials, particularly their sensitivity in the early and preclinical stages of the disease, which hampers their reliability as indicators of AD progression.
- The Functional Activities Questionnaire (FAQ) demonstrated the most substantial weighted mean change over 12 weeks, followed by the Mini‐Mental State Examination (MMSE).
- Functional measures outperformed other measure categories.
- Composite scores of integrated Alzheimer's Disease Rating Scale and Clinical Dementia Rating‐Sum of Boxes are more sensitive to change than their individual cognitive components, possibly driven by the functional components of the score.
- Neuropsychiatric measures analyzed in this study appeared to be the least sensitive in measuring progression.

## 1 INTRODUCTION

As treatments for Alzheimer's disease (AD) were sought and developed, there was a need to have instruments that could accurately measure clinical progression in AD. Some measures that had been initially developed as clinical screening tools served this purpose, for example, the Mini‐Mental State Examination (MMSE). Other measures were specifically designed to measure AD progression.

A key aim of clinical trials is to reduce or halt the rate of clinical progression of AD. Many trials have been unsuccessful, but we now have the first trials that have shown clinical benefit as well as an effect on the underlying pathology being targeted.^1^, ^2^ In turn, this has led to the first full US Food and Drug Administration (FDA)‐approved treatment.^3^ To determine the effectiveness of treatments, there must be robust and reliable tools for measuring AD progression. For at least 30 years there has been recognition that the measures of progression used in AD clinical trials are problematic.^4^ One criticism is that clinical measures of progression are affected by symptomatic treatments (eg, cholinesterase inhibitors [ChEIs]) and therefore, the true disease‐modifying effects of potential therapies are masked.^5^ Another, more significant, concern is that the most commonly used measures of clinical progression are potentially not sensitive enough in early and preclinical stages of AD and so are not reliable indicators of AD progression.^6^ The disease heterogeneity, particularly in trials prior to biomarker enrichment, will also play a role. These criticisms have led to the development of newer composite measures as endpoints in clinical trials.^7^ One of the issues, though, is the lack of a standard adopted composite score in AD clinical trials and therein the proliferation of many different composite measures of AD progression. This makes comparison between studies very difficult.

This has led to a focus on biomarkers that can act as surrogate outcome markers in therapeutic trials. This shift in focus has been seen most clearly in the case of anti‐amyloid therapies that act to reduce amyloid load in the brain. However, as highlighted by the controversy around the clinical utility of these therapeutic agents,^8^ it is not clear that amyloid reduction, or a statistically significant change on a clinical rating scale, is linked to meaningful clinical improvement.^9^ Indeed, clinical trial participants in whom amyloid plaque reduction has been demonstrated still experience progressive neurodegeneration.^10^ Much of the difficulty in trying to develop measures of progression in AD stems from the clinical and pathological heterogeneity of the disease. There are diverse sources of this difficulty, including, but not limited to, genetics, neuropathology, and demographics.^11^ There has been a subsequent drive to enrich trial designs to account for these factors.

### 1.1 Rationale and aims of the review

Many clinical trials are being terminated for futility; however, no clear quantification of the sensitivity of progression measures used in clinical trials exists that adequately proves the ability of an instrument to detect change over the duration of a trial, even in placebo patients. It is not known quantitatively whether some clinical progression measures perform better than others in AD clinical trials. This performance rests on the sensitivity of a measure to detect change over time and whether this change is clinically meaningful. By analyzing the placebo groups of clinical trials, which have confirmed positive amyloid (Aβ+) pathology in their included participants, this review aims to compare the sensitivity of progression measures at detecting change and to indicate whether particular progression measures are better placed than others to be utilized in AD clinical trials.

## 2 METHODS

### 2.1 Search strategy and selection criteria

A search of the International Prospective Register of Systematic Reviews (PROSPERO) and the Cochrane Library revealed that no similar review had been undertaken.

The research question was formulated ahead of the search strategy. The Population, Intervention, Control, Outcomes, and Study Design principle (PICOS) informed this.

#### 2.1.1 Population

The study population comprised males and females over 18 years of age with a diagnosis of AD, mild cognitive impairment (MCI) due to AD pathology, or prodromal AD in whom AD pathology had been indicated by evidence of Aβ deposition on amyloid positron emission tomography (PET) imaging or through cerebrospinal fluid (CSF) analysis prior to inclusion in a clinical trial. Only placebo groups were used in the review.

#### 2.1.2 Intervention

Studies investigating therapeutic agents for the treatment of AD whether in established AD or prodromal AD or MCI due to AD pathology. Therapeutic interventions related only to cognitive decline.

#### 2.1.3 Control

Not applicable – the placebo groups in the therapeutic trials formed the target population for this review.

#### 2.1.4 Outcomes

The assessment of progression in AD via novel clinical progression measures/models or established clinical measures.

#### 2.1.5 Study design

Randomized controlled trials (RCTs) were exclusively sought.

**FIGURE 1 alz14314-fig-0001:**
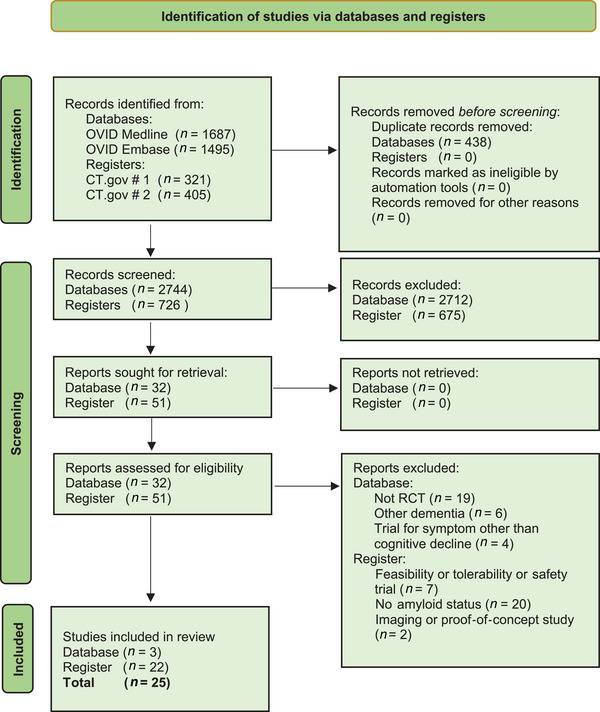
PRISMA flow diagram.^44^

This process refined the research question underpinning the systematic review. Figure 1 provides a visual summary of the procedure for selecting studies for inclusion in the systematic review. For further details on adherence to PRISMA guidelines, please refer to Table S1.

### 2.2 Resources for search

The following resources were used for the study search: MEDLINE (Ovid interface), Embase (OVID interface), PubMed, Google Scholar, National Institutes of Health ClinicalTrials.gov, The Australian Imaging, Biomarker and lifestyle flagship study's list of publications, International Clinical Trials Registry Platform (ICTRP World Health Organization), International Standard Randomised Controlled Trial Number registry (ISRCTN), European Union Drug Regulating Authorities Clinical Trials Database (EudraCT), and Researchregistry.com. Search terms and output statistics are given in Sections 1 and 2 of Appendix I in the Supplementary materials, while search question definitions can be found in Section 3 of the same file.

**RESEARCH IN CONTEXT**

1. **Systematic review**: By analyzing the placebo groups of clinical trials, which confirmed amyloid beta‐positive (Aβ+) pathology in their included participants, this analysis aims to compare the sensitivity of progression measures at detecting change and to suggest which progression measures are better placed than others to be utilized in Alzheimer's disease (AD) clinical trials.
2. **Interpretation**: Functional measures can detect change in early disease states, and their inclusion in composite measures enhances the sensitivity to detect clinical progression.
3. **Future directions**: The weighted mean change for the outcome measures included in this analysis are reasonable estimates of progression in Aβ+ enriched AD randomized controlled trials. We analyzed the most used AD trial outcome measures. Further analysis will detail how measures change during different time periods. For example, it is not clear which measures pick up change in the first 12 months of follow‐up and which detect greater change past this time point.

### 2.3 Inclusion and exclusion criteria

Studies were included if they met the defined criteria as follows:[Fig alz14314-fig-0001]

1. RCT in design and trialing a therapeutic intervention.
2. RCTs that used clinical outcome/progression measures.
3. Participant inclusion criteria stipulated that only Aβ+ subjects/subjects with elevated Aβ were included in the trial as determined by either amyloid PET or CSF analysis.
4. Subjects had a diagnosis of AD or were cognitively normal (CN) but with evidence of Aβ deposition.
5. The RCT must have a placebo group and have reported data on clinical outcome/progression measures for the placebo group. In circumstances where the RCT did not have a published report in a peer‐reviewed journal, it could be included if data were reported on a recognized forum such as ClinicalTrials.gov.

Studies were excluded if they met the defined criteria as follows:

1. Observational, case study or reports, case‐control or cohort study in design
2. RCTs reporting elevated tau levels but in which there is no account of Aβ+
3. RCTs that included patients with other forms of dementia/pathology, for example, suspected cerebral vascular disease
4. RCTs that trailed an intervention for symptoms/difficulties arising other than cognitive decline
5. RCTs that trailed an intervention aimed at caregivers, for example, education intervention
6. Insufficient data were reported, for example, no data on change in clinical measures from baseline to endpoint or idiosyncratic and sparse clinical measures used.

The inclusion and exclusion criteria are highly objective, and thus, the reviewer's assessment was deemed adequate. No limits were placed on the number of trial participants, purported severity of AD, trial length, class of therapeutic agent, main trial outcome, and the number of clinical outcome/progression measures used. The details of each RCT are reported in Table S2 Appendix I of the Supplementary materials.

All of the included trials involved disease‐modifying compounds in the active arm(s) of the RCT. Of the included RCTs, only six included the prescription of a ChEI as an exclusion criterion.^1^, ^12^, ^13^, ^14^, ^15^, ^16^

The large majority of the included studies appeared to use the National Institite of Nurological and Communicative Disorders and Stroke/Alzheimer's Disease and Related DA (1984) (NINCDS/ADRDA), National Institute on Aging‐Alzheimer's Association (2011) (NIA‐AA), and International Working Group (2014) (IWG) diagnostic terminologies. This was somewhat inconsistent, as “probable AD” was mentioned alongside “prodromal AD,” indicating the use of terms from two different nosologies. Largely, the use of such terms implied a clinical diagnosis but with the added element that all participants had confirmed Aβ+ status prior to trial enrollment. Therefore, in determining whether a biological or clinical definition of AD was used, it appears that a combination of both was employed. See Table S3, Section 8, Appendix I of the Supplementary materials for a description of the terms used.

### 2.4 Data extraction process

Data, including baseline characteristics for placebo group participants, for each outcome measure (if available in two or more RCTs), were manually extracted from either the published study or the data published in the registry entry on ClinicalTrials.gov. Studies reported either arithmetic mean (AM) change in an outcome measure or least‐squares mean (LSM) with the corresponding standard error (SE), standard deviation (SD), or 95% confidence interval (CI).

From the 25 included studies there were 26 data sets for analysis. A possible 27th data set was requested from a corresponding author for the Marguerite RoAD trial,^12^ but no response was received.

In line with best practices, as set out in Cochrane guidelines,^17^ risk of bias (RoB) in the included RCTs was assessed using the revised tool to assess RoB in randomized trials.^18^ Full details can be found in Section 1 of Appendix II. Figures S1 and S2 in the indicated section represent the RoB in studies that passed our criteria.

### 2.5 Outcome measures

Twelve outcome measures used in the trials that passed the detailed inclusion/exclusion criteria are detailed in Table 1.

**TABLE 1:** List of outcome measures assessed.

Abbreviation	Measure name
ADAS‐Cog‐11	Alzheimer's Disease Assessment Scale‐Cognitive Subscale 11
ADAS‐Cog‐13	Alzheimer's Disease Assessment Scale‐Cognitive Subscale 13
ADAS‐Cog‐14	Alzheimer's Disease Assessment Scale‐Cognitive Subscale 14
ADCOMS	Alzheimer's Disease Composite Score
ADCS ADL‐PI	Alzheimer's Disease Cooperative Study‐Activities of Daily Living‐Prevention Instrument
ADCS^_^ADL‐MCI	Alzheimer's Disease Cooperative Study‐Activities of Daily Living for Mild Cognitive Impairment
ADCS‐ADL	Alzheimer's Disease Cooperative Study‐Activities of Daily Living Inventory
ADCS‐iADL	Alzheimer's Disease Cooperative Study‐Activities of Daily Living inventory, instrumental items
APCC	Alzheimer's Prevention Initiative Preclinical Composite Cognitive test
CDR‐SB	Clinical Dementia Rating Scale Sum of Boxes
ECOG	Measurement of Everyday Cognition
EQ‐5D	EuroQol 5‐dimension questionnaire
FAQ	Functional Activities Questionnaire
iADL	Instrumental Activities of Daily Living
iADRS	Integrated Alzheimer's Disease Rating Scale
MMSE	Mini‐Mental State Examination
NPI	Neuropsychiatric Inventory
NTB	Neuropsychological Test Battery
QOL‐AD	Quality of Life in Alzheimer's Disease
RBANS	Repeatable Battery for the Assessment of Neuropsychological Status
ZCI‐AD	Zarit Caregiver Interview for Alzheimer's Disease

### 2.6 Statistical analysis

Statistical analyses were performed in R version 4.2.0^19^ using the metafor package,^20^, ^21^ which was updated to include inverse variance to allow for the inclusion of heterogeneous studies in meta‐analyses.

As well as being a reasonable period over which progression effects may be observable, a period of 12 weeks was chosen to maximize available trial data. The mean change in the outcome measure per 12 weeks was calculated for the data according to the following formula:

$$\mathrm{Weighted}\;\mathrm{mean}\;\mathrm{change}=ic\;i\left(\frac{t}{12}\times r\right),$$

where *c* denotes a change in LSM or AM, *t* the total trial duration in weeks, and *r* the score range for a given outcome measure.

Due to the expected high heterogeneity of outcome measures, that is, LSM versus AM, we opted for a random effects model with an inverse variance heterogeneous meta‐analysis known as IVhet.^22^

Moderator variables were also assessed and included the mean age of placebo participants at baseline, the percentage of females, and AD stage targeted as ascertained by the mean MMSE score at baseline. It was felt that all three factors could reasonably be expected to influence the weighted mean change in outcome measures. Thus, meta‐regressions were performed with the percentage of female, mean age at baseline, and AD stage when variation existed. The emmeans package was used to predict the estimated value of mean change under a model that assumed the mean of the continuous moderator and the mean of the dummy variables for each level of the categorical moderator.

## 3 RESULTS

Twenty‐five RCTs and 12 measures were included. The included trials spanned a publication period of 2015 to 2022. Fifteen trials had trial registration, published trial data on ClinicalTrials.gov, and a peer‐reviewed linked publication in a journal. The characteristics of the placebo groups are summarized in Table 2.

**TABLE 2:** Summary of baseline characteristics of placebo groups in included studies. In the absence of registered trial names or multiple companies, the first author of the paper where the results were published is given as the study name.

Study name	NCT number	Placebo (*N*)	Females (%)	Age (SD)	MMSE	Diagnosis
AMARANTH 2019	NCT02245737	740	53.8	71.4 (6.9)	20 to 30 inclusive	Probable AD or MCI due to AD NIA‐AA
APECS 2019	NCT01953601	485	43.9	71.6 (7.1)	≥24	Prodromal AD
BEAT‐AD 2016	NCT01782742	4	75	78.1 (8.0)	10 to 20 inclusive	Probable AD NINCDS/ADRDA
Coric 2015	NCT00890890	131	42	71.6 (7.8)	24 to 30 inclusive	Prodromal AD
CREAD2 2020	NCT03114657	399	56.4	70.7 (7.9)	≥22	Probable or Prodromal AD NIA‐AA
CREAD 2020	NCT02670083	409	60.40	70.3 (8.4)	≥22	Probable or Prodromal AD NIA‐AA
DAYBREAK 2019	NCT02783573	562	61.9	72.1 (7.1)	20 to 26 inclusive	Probable AD or MCI due to AD NIA‐AA
Eli Lilly 2018	NCT01561430	20	55	67.73 (7.1)	20 to 26 probable AD 27 to 30 MCI due to AD	MCI due to AD or probable AD NINCDS/ADRDA
EMERGE 2021	NCT02484547	548	52.90	70.8 (7.4)	24 to 30 inclusive	MCI due to AD or mild AD NIA‐AA
ENGAGE 2021	NCT02477800	545	52.70	69.8 (7.7)	24 to 30 inclusive	MCI due to AD or mild AD NIA‐AA
EXPEDITION 3 2018	NCT01900665	1072	58.9	73.26 (8.0)	20 to 26 inclusive	Probable AD NINCDS/ADRDA
Frolich 2019	NCT02240693	43	65.1	72.2 (6.5)	≥24	Prodromal AD
Mission AD 1 & 2 2021	NCT02956486	1108	53.6	72.1 (7.1)	≥24	MCI due to AD or mild AD NIA‐AA
Mullins 2019	NCT01255163	14	60	74.0 (6.4)	>20	MCI or early AD
NAVIGATE‐AD 2021	NCT02791191	133	58	72.54 (7.8)	20 to 26 inclusive	Mild AD NIA‐AA
Novartis 2021	NCT03131453	456	63.2	65–70	≥24	Cognitively unimpaired
Potter 2021	NCT01409915	20	55	70.15 (6.4)	10 to 26 inclusive	Moderate AD MMSE criteria
REVERSE‐SD 2021	NCT03402659	83	51.8	72.6 (NA)	20 to 28 inclusive	Mild AD
SCarlet RoAD 2021	NCT01224106	266	56	69.5 (7.5)	≥24	Prodromal AD NINCDS/ADRDA
Sperling 2021	NCT02569398	185	58.40	70.2 (5.8)	NA	Clinically normal
Swanson 2021	NCT01767311	238	59	71 (NA)	22 to 30 inclusive	MCI due to AD or mild AD NIA‐AA
Teng 2022	NCT03289143	135	55.6	69.7 (7.3)	≥20	Probable or prodromal AD NIA‐AA
Trailblazer‐ALZ 2021	NCT03367403	126	51.6	75.4 (5.4)	20 to 28 inclusive	Prodromal AD
van Dyck 2016	NCT01227564	21	42.9	69.6 (6.8)	≥25	Subjective memory complaint and no dementia diagnosis
van Dyck 2019	NCT02167256	80	38.8	>55	18 to 26 inclusive	Probable AD NIA‐AA

Abbreviations: AD, Alzheimer's disease; MCI, mild cognitive impairment; MMSE, mini‐mental state examination; NIA‐AA, National Institute on Aging‐Alzheimer's Association; NINCDS/ADRDA, National Institite of Nurological and Communicative Disorders and Stroke/Alzheimer's Disease and Related DA; SD, standard deviation.

Several progression measures were excluded due to their being used in two or fewer studies. These were Alzheimer's Prevention Initiative Preclinical Composite Cognitive test, Everyday Cognition, EuroQol 5‐dimension questionnaire, Instrumental Activities of Daily Living, Repeatable Battery for the Assessment of Neuropsychological Status, Zarit Caregiver Interview for Alzheimer's Disease, Alzheimer's Disease Cooperative Study‐Activities of Daily Living‐Prevention Instrument, and Alzheimer's Disease Composite Score.

In the preliminary analysis, an assessment was made of the heterogeneity of variance across studies for each cognitive outcome measure. The I^2^ statistic and Q‐statistic were used in this analysis. This showed high heterogeneity of variance across studies for nearly all the selected measures, for example, Clinical Dementia Rating‐Sum of Boxes (CDR‐SB) (I^2^ 91.62% and Q‐statistic 202.44, *p* < .0001) and MMSE (I^2^ 86.7 and Q‐statistic 112.7, *p* < .0001). Only one measure appeared to have low heterogeneity of variance across studies (six studies), ADCS‐ADL (I^2^ 0% and Q‐statistic 3.22, *p* = .665).

Given the high heterogeneity between the studies, a random‐effects model^23^ was utilized for the main meta‐regression to account for this. This model assumes that different studies estimate different effects but that these are related, with a resultant adjustment to study weighting according to the extent of heterogeneity, or variation, among the effects.^17^ The random‐effects model was further adjusted using inverse variance to assign weights to each study. The specific models for each outcome were dictated by the availability of data on the covariates and heteromorphism in the covariates. These can be found in Table S4, Appendix III of the Supplementary materials.

Alzheimer's Disease Cooperative Study‐Activities of Daily Living for Mild Cognitive Impairment (ADCS‐ADL‐MCI) showed no variation in AD stage across the studies utilizing the tool, so only the percentage of females and mean age were regressed. Neither Quality of Life in Alzheimer's Disease (QOL‐AD) nor Alzheimer's Disease Assessment Scale‐Cognitive Subscale 14 (ADAS‐Cog‐14) was used in sufficient studies to enable correcting for all three variables. Upon assessment, it was decided that the mean age at baseline and percentage of females at baseline for the studies did not differ significantly, and therefore only the AD stage was regressed. The remaining nine measures underwent correction for all three modifiers. Table S5 of Appendix III in the Supplementary materials lists outcome measures by weighted mean change over 12 weeks. A comparison of raw weighted mean change and covaried mean change shows the necessity of correcting for age, gender, and AD stage.

The results of the meta‐analysis for individual progression measures are detailed in Figure 2. In each panel, a group of measures is meta‐regressed and displayed below each group. It can be seen from the meta‐regressions that functional measures outperform cognitive and composite measures, while neuropsychiatric batteries barely detect any change in the brief period assessed. Individual forest plots for each meta‐regression are presented in Figures S3–S14, Appendix IV of Supplementary materials.

**FIGURE 2:**
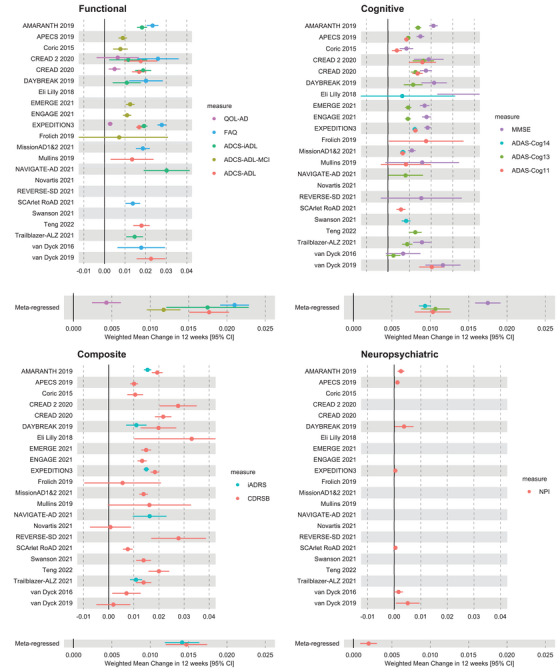
Forest plots of meta‐regression for functional, cognitive, composite, and neuropsychiatric measures from 25 RCTs. Each panel depicts one group of progression measures with their respective meta‐regression results displayed under the panel for clarity.

## 4 DISCUSSION

We performed meta‐analyses of cognitive, functional, composite, and neuropsychiatric measures used in RCTs, including only placebo arms with Aβ+ subjects. While all disease stages were covered, most studies targeted early and prodromal AD phases. We found that functional measures, contrary to previous publications,^24^ specifically measures assessing Activities of Daily Living (ADLs), either alone or in composite scores, outperformed other measures. The Functional Activities Questionnaire (FAQ), a 10‐point functional measure, showed the largest weighted percentage mean change over 12 weeks (2.12%, 95% CI: 1.9 to 2.3). The FAQ was previously validated as reliable in differentiating between normal controls, MCI, and mild dementia.^25^

The FAQ, administered solely to informants, may exhibit variability as more educated or cohabiting informants tend to score impairments more severely.^26^ It is crucial to consider the functionalities of FAQ rates. ADLs cover basic self‐care like bathing, while Instrumental Activities of Daily Living (IADLs) involve complex tasks like managing finances, medications, and shopping. The FAQ emphasizes IADLs, with evidence showing that impairment in these skills correlates with amyloid burden, even in MCI patients,^27^ and declines early in AD.^28^ The CDR‐SB, mainly informant‐based, includes IADLs and ADLs across three domains, plus three cognitive domains, of which two are assessed in patients. The greater emphasis on IADLs in the FAQ may explain its superior performance. Both FAQ and CDR‑SB have recently been used to assess early AD progression,^29^ with our results supporting their sensitivity.

The ADCS‐ADL and its instrumental subscale, the ADCS‐iADL, performed very similarly (Appendix III). They appear to be good measures for detecting progression. Notably, they outperformed the ADCS‐MCI modification, specifically calibrated to pick up progression in MCI subjects.

While four out of five of our functional measures exceeded the performance of all other measures, the QoL‐AD appears to be an exception (0.36% CI 0.16% to 0.60%). The QoL‐AD is administered to the subject and contains a mix of neuropsychiatric, social, relationship, and functional items. Potentially, the performance of the QOL‐AD is attenuated by the inclusion, for example, of neuropsychiatric symptoms, uncommon in early AD. This would also explain the inferior performance of the neuropsychiatric inventory (NPI) demonstrated in this analysis. It was previously assumed that measures with a larger scale were more likely to detect small amounts of change; however, both in the case of NPI and iADRS we saw that this was irrelevant. This highlights the need for clinical measures to be targeted at the symptoms experienced at various stages of the disease. This also shows that other factors, such as the study population, play a key role in influencing the appropriateness of an instrument for measuring progression in AD.

The ADAS‐Cog, and its various versions, are reported to be the most used primary outcome instrument in AD clinical trials.^30^ However, they have been criticized for having low sensitivity to detect change and significant ceiling effects in mildly or minimally impaired cohorts.^31^ This has also been highlighted in predementia cohorts^32^ and was consistent with our findings above. Most notably, ADAS‐Cog‐14 appears to be the least sensitive to change and is the third least sensitive measure of progression examined in this study. Other measures, such as the CDR‐SB, were said to be better in these patient groups, with one head‐to‐head analysis of ADAS‐Cog and CDR‐SB showing the latter to be better at detecting treatment differences in AD clinical trials.^33^ This work, unlike ours, did not focus on RCTs where Aβ+ status was an inclusion criterion, a significant limitation, and did not correct for possible modifiers of effect. In our findings, all three versions of the ADAS‐Cog displayed inferior ability to measure progression compared to the CDR‐SB. It has been shown that ADAS‐Cogs perform better in composite scores if functional items, including FAQ are added,^34^ again consistent with our observation of the utility of FAQ.

There has been criticism of the proliferation of composite scores in AD trials and that many are not validated. Specifically, criticism has focused on the lack of available data on the performance of the individual components of composite scores.^35^ It has been suggested that composite scores, including the iADRS, may outperform their components when determining effect sizes of interventions.^36^ Among our composite progression measures, the iADRS outperformed one of its components, the ADAS‐Cog‐14, but not the other, the ADCS‐iADL.

Of the purely cognitive measures, the MMSE performed better than expected despite suspected ceiling effects,^37^ which are affected by education levels.^38^ It is likely that the pooled placebo subjects in our study had higher levels of education, as was shown across previous AD trials.^39^ Ceiling effects may be reinforced as a result. Unfortunately, educational attainment was not available across all RCTs to allow for the correction of such effects.

There are, of course, desirable features of progression measures other than greater sensitivity in detecting change, such as the dynamic range of the measure, practical considerations such as acceptability to the subject and informant, and time constraints. In this last respect, a scale such as the CDR has a longer duration of administration than a measure such as the FAQ or ADCS‐ADL, which take 15 to 30 min to complete.^40^ Such factors must be considered before choosing measures.

The results are subject to potential limitations. Every effort was made to find trials for inclusion, but there may be unincluded data sets. The quality of studies included was rigorously assessed using the Cochrane RoB tool, and this has shown some to be at RoB (Figure S2, Appendix II).

The RCTs assume a linear change in the progression measure, as shown by the reporting of mean change since baseline. We are aware that this will distort sensitivity in progression measures where a non‐linear trajectory exists. This, in fact, may be the case for tests such as MMSE; however, we cannot deduce the true trajectory based on publicly available data. This highlights the need for more open sharing of data across clinical trials and academic communities.

The inclusion of cognitively unimpaired subjects must be noted. Only three studies^13^, ^14^, ^15^ reported enrolling participants with Clinical Dementia Rating Global Scores (CDR‐GS) of 0, including one where some participants had a MMSE score of 24 or higher, despite being labeled “cognitively unimpaired.” Two of these described participants as “clinically normal” or “cognitively unimpaired,” while the third included both CDR‐GS of 0 and 0.5 under “prodromal AD.” These studies involved 684 participants, representing less than 10% of the subjects in the meta‐analysis. This figure is likely to be an overestimate.

Definitions of “cognitively unimpaired” and “prodromal AD” vary. One study^14^ classified “cognitively unimpaired” participants as those with at least one APOE ε4 gene and being Aβ+. In other contexts, such individuals might be described as “asymptomatic at risk with AD pathology” per the IWG (2014) classification, or as having “prodromal AD.” Despite different terminology, most participants had cognitive impairment and were Aβ+. Therefore, while diagnostic terms varied, the core features of cognitive impairment and amyloid positivity remained consistent across studies. Clinical diagnostic criteria were still the primary means of diagnosing AD. This reliance on clinical criteria, despite biomarker confirmation, highlights the gap between research and clinical practice. The continued use of traditional clinical assessments underscores the complexity of Alzheimer's diagnosis and the need to integrate biomarker data into clinical frameworks more comprehensively.

Analyzing placebo patients potentially leads to placebo effects in the pooled cohort. These have already been described in neuropsychiatric symptoms in AD trial participants.^41^ Additionally, some patients may have been taking ChEIs. Of the included RCTs, only six included prescription of a ChEI as an exclusion criterion.^1^, ^12^, ^13^, ^14^, ^15^, ^16^ This constitutes 14% of the placebo subjects in this analysis and reflects the pragmatic recruitment of some RCTs in this area. Furthermore, the sensitivities we report here may differ, albeit marginally, in the treatment arm of the trials, and this study merely addresses the foundational steps required to assess progression measures.

The choice of 12 weeks for progression assessment was a pragmatic decision to maximize data incorporation. Our results, when considered over 12 months, are in line with reported minimally clinically important differences (MCID) in the outcome measures.^42^

Additional moderator analyses such as educational attainment or inter‑rater variability would be relevant, but lack of data across all studies and heterogeneity in trial methodology prevented this. We believe that the moderators chosen were the most feasible with the given data and likely to have the most modifying effects on estimates.

Our results support the notion that functional difficulties are present in early AD and MCI, as other work has shown,^43^ and that they can be identified with standard outcome measures like the FAQ. Furthermore, progression measures that rely solely on cognitive or neuropsychiatric domains are not ideal for use in AD trials that target mild or early AD. In such cases, it is advisable to use measures that include cognitive and complex functional assessments. Future studies should assess correlations between these findings in RCTs and other cohorts such as observational cohorts.

## CONFLICT OF INTEREST STATEMENT

The authors declare no conflicts of interest. Author disclosures are available in the Supporting information.

## CONSENT STATEMENT

While the participants of each RCT used in this study provided consent for the given study, all data secured for the meta‐analysis are publicly available summary data, and therefore no consent was required for their use.

## Supporting information



Supporting Information

Supporting Information

## References

[1] Mintun MA , Lo AC , Duggan Evans C , et al. Donanemab in early Alzheimer's disease. N Engl J Med. 2021;384(18):1691‐1704. 33720637 10.1056/NEJMoa2100708

[2] van Dyck CH , Swanson CJ , Aisen P , et al. Lecanemab in early Alzheimer's disease. N Engl J Med. 2023;388(1):9‐21. 36449413 10.1056/NEJMoa2212948

[3] Harris E . Alzheimer Drug Lecanemab Gains Traditional FDA Approval. JAMA. 2023;330(6):495. 10.1001/jama.2023.12548 37466999

[4] Swash M , Brooks DN , Day NE , Frith CD , Levy R , Warlow CP . Clinical trials in Alzheimer's disease. A report from the Medical Research Council Alzheimer's Disease Clinical Trials Committee. J Neurol Neurosurg Psychiatry. 1991;54(2):178‐181. 1843446 10.1136/jnnp.54.2.178 PMC1014359

[5] Mcghee DJM , Ritchie CW , Thompson PA , Wright DE , Zajicek JP , Counsell CE . A systematic review of biomarkers for disease progression in Alzheimer's disease. PLoS One. 2014;9(2):e88854. 24558437 10.1371/journal.pone.0088854 PMC3928315

[6] Cummings JL , Doody R , Clark C . Disease‐modifying therapies for Alzheimer disease: challenges to early intervention. Neurology. 2007;69(16):1622‐1634. 17938373 10.1212/01.wnl.0000295996.54210.69

[7] Vellas B , Bateman R , Blennow K , et al. Endpoints for pre‐dementia AD trials: a report from the EU/US/CTAD task force. J Prev Alzheimers Dis. 2015;2(2):128‐135. 26247004 10.14283/jpad.2015.55 PMC4523051

[8] Thambisetty M , Howard R . Lecanemab trial in AD brings hope but requires greater clarity. Nat Rev Neurol. 2023;19(3):132‐133. 36609712 10.1038/s41582-022-00768-w

[9] Mead S , Fox NC . Lecanemab slows Alzheimer's disease: hope and challenges. Lancet Neurol. 2023;22(2):106‐108. 36681438 10.1016/S1474-4422(22)00529-4

[10] Ackley SF , Zimmerman SC , Brenowitz WD , et al. Effect of reductions in amyloid levels on cognitive change in randomized trials: instrumental variable meta‐analysis. BMJ. 2021;372:n156. 33632704 10.1136/bmj.n156 PMC7905687

[11] Duara R , Barker W . Heterogeneity in Alzheimer's Disease diagnosis and progression rates: implications for therapeutic trials. Neurotherapeutics. 2022;1:8‐25. 10.1007/s13311-022-01185-z PMC9130395 35084721

[12] Klein G , Delmar P , Voyle N , et al. Gantenerumab reduces amyloid‐β plaques in patients with prodromal to moderate Alzheimer's disease: a PET substudy interim analysis. Alzheimers Res Ther. 2019;11(1):101. 31831056 10.1186/s13195-019-0559-z PMC6909550

[13] Sperling R , Henley D , Aisen PS , et al. Findings of efficacy, safety, and biomarker outcomes of atabecestat in preclinical Alzheimer disease: a truncated randomized phase 2b/3 clinical trial. JAMA Neurol. 2021;78(3):293‐301. 33464300 10.1001/jamaneurol.2020.4857 PMC7816119

[14] NCTs 17‐00718, A randomized, double‐blind, placebo‐controlled, parallel group study to evaluate the efficacy and safety of CNP520 in participants at risk for the onset of clinical symptoms of Alzheimer's Disease (Generation 2). 2017.

[15] Van Dyck C , Sadowsky C , Le Prince Leterme G , et al. Vanutide Cridificar (ACC‐001) and QS‐21 adjuvant in individuals with early Alzheimer's disease: amyloid imaging positron emission tomography and safety results from a phase 2 study. J Prev Alzheimers Dis. 2016;3(2):75‐84. 29210443 10.14283/jpad.2016.91

[16] Frölich L , Wunderlich G , Thamer C , Roehrle M , Garcia M Jr , Dubois B . Evaluation of the efficacy, safety and tolerability of orally administered BI 409306, a novel phosphodiesterase type 9 inhibitor, in two randomised controlled phase II studies in patients with prodromal and mild Alzheimer's disease. Alzheimers Res Ther. 2019;11(1):18. 30755255 10.1186/s13195-019-0467-2 PMC6371616

[17] Higgins JPT , Thomas J , Chandler J , et al. Cochrane Handbook for Systematic Reviews of Interventions. John Wiley & Sons; 2019.

[18] Sterne JAC , Savović J , Page MJ , et al. RoB 2: a revised tool for assessing risk of bias in randomised trials. BMJ. 2019;366. 10.1136/bmj.l4898 31462531

[19] R‐Core T . A Language and Environment for Statistical Computing. R Foundation for Statistical Computing; 2022.

[20] Viechtbauer W . Conducting Meta‐Analyses in R with the metafor Package. J Stat Softw. 2010;36(3):1‐48.

[21] Lenth R . Emmeans: Estimated Marginal Means, Aka Least‐Squares Means. R Package; 2024. Version 1.7. 0; 2021. https://rvlenth.github.io/emmeans/

[22] Doi SAR , Barendregt JJ , Khan S , Thalib L , Williams GM . Advances in the meta‐analysis of heterogeneous clinical trials I: the inverse variance heterogeneity model. Contemp Clin Trials. 2015;45:130‐138. 26003435 10.1016/j.cct.2015.05.009

[23] DerSimonian R , Laird N . Meta‐analysis in clinical trials. Control Clin Trials. 1986;7(3):177‐188. 3802833 10.1016/0197-2456(86)90046-2

[24] Samtani M , Raghavan N , Novak G , Nandy P , Narayan VA . Disease progression model for Clinical Dementia Rating‐Sum of Boxes in mild cognitive impairment and Alzheimer's subjects from the Alzheimer's Disease Neuroimaging Initiative. Neuropsychiatr Dis Treat. 2014;10:929‐952. 24926196 10.2147/NDT.S62323 PMC4049432

[25] González DA , Gonzales MM , Resch ZJ , Sullivan AC , Soble JR . Comprehensive evaluation of the Functional Activities Questionnaire (FAQ) and its reliability and validity. Assessment. 2021;29(4):748‐763. 33543638 10.1177/1073191121991215 PMC8339133

[26] Hackett K , Mis R , Drabick DAG , Giovannetti T . Informant reporting in mild cognitive impairment: sources of discrepancy on the Functional Activities Questionnaire. J Int Neuropsychol Soc. 2020;26(5):503‐514. 31964443 10.1017/S1355617719001449 PMC7205566

[27] Marshall GA , Olson LE , Frey MT , et al. Instrumental activities of daily living impairment is associated with increased amyloid burden. Dement Geriatr Cogn Disord. 2011;31:443‐450. 21778725 10.1159/000329543 PMC3150869

[28] Perneczky R , Pohl C , Sorg C , et al. Impairment of activities of daily living requiring memory or complex reasoning as part of the MCI syndrome. Int J Geriatr Psychiatry. 2006;21:158‐162. 16416470 10.1002/gps.1444

[29] Chandler J , Georgieva M , Desai U , et al. Impact of differential rates of disease progression in amyloid‐positive early Alzheimer's disease: findings from a longitudinal cohort analysis. J Prev Alzheimers Dis. 2024;11:320‐328. 38374738 10.14283/jpad.2024.28

[30] Connor DJ , Sabbagh MN . Administration and scoring variance on the ADAS‐Cog. J Alzheimer's Dis. 2008;15:461‐464. 18997299 10.3233/jad-2008-15312 PMC2727511

[31] Cano SJ , Posner HB , Moline ML , et al. The ADAS‐cog in Alzheimer's disease clinical trials: psychometric evaluation of the sum and its parts. J Neurol Neurosurg Psychiatry. 2010;81(12):1363‐1368. 20881017 10.1136/jnnp.2009.204008

[32] Kueper JK , Speechley M , Montero‐Odasso M . The Alzheimer's Disease Assessment Scale‐Cognitive Subscale (ADAS‐Cog): modifications and responsiveness in pre‐dementia populations. A narrative review. J Alzheimer's Dis. 2018;63:423‐444. 29660938 10.3233/JAD-170991 PMC5929311

[33] Wessels AM , Dowsett S , Sims J . Detecting treatment group differences in Alzheimer's disease clinical trials: a comparison of Alzheimer's disease assessment scale‐cognitive subscale (ADAS‐Cog) and the Clinical Dementia Rating‐Sum of Boxes (CDR‐SB). J Prev Alzheimers Dis. 2018;5(1):15‐20.29405227 10.14283/jpad.2018.2

[34] Raghavan N , Samtani MN , Farnum M , et al. The ADAS‐Cog revisited: novel composite scales based on ADAS‐Cog to improve efficiency in MCI and early AD trials. Alzheimers Dement. 2013;9(1):S21. 23127469 10.1016/j.jalz.2012.05.2187 PMC3732822

[35] Schneider LS , Goldberg TE . Composite cognitive and functional measures for early stage Alzheimer's disease trials. Alzheimers Dement (Amst). 2020;12(1):e12017. 32432155 10.1002/dad2.12017 PMC7233425

[36] Liu‐Seifert H , Andersen S , Case M , et al. Statistical properties of continuous composite scales and implications for drug development. J Biopharm Stat. 2017;27(6):1104‐1114. 28402165 10.1080/10543406.2017.1315819

[37] Franco‐Marina F , García‐González JJ , Wagner‐Echeagaray F , et al. The Mini‐Mental State Examination revisited: ceiling and floor effects after score adjustment for educational level in an aging Mexican population. Int Psychogeriatr. 2010;22(1):72‐81. 19735592 10.1017/S1041610209990822

[38] Murden RA , Mcrae TD , Kaner S , Bucknam ME . Mini‐Mental State Exam scores vary with education in blacks and whites. J Am Geriatr Soc. 1991;39(2):149‐155. 1991947 10.1111/j.1532-5415.1991.tb01617.x

[39] Franzen S , Smith JE , van den Berg E , et al. Diversity in Alzheimer's disease drug trials: the importance of eligibility criteria. Alzheimer's & Dementia. 2022;18(4):810‐823. 10.1002/alz.12433 PMC8964823 34590409

[40] Fish J . Alzheimer's Disease Cooperative Study ADL Scale. Springer New York; 2011:111‐112.

[41] Zhang N , Lv Y , Li H , et al. Quantifying placebo responses in clinical evaluation of neuropsychiatric symptoms in Alzheimer's disease. Eur J Clin Pharmacol. 2019;75(4):497‐509. 30612155 10.1007/s00228-018-02620-x

[42] Andrews JS , Desai U , Kirson NY , Zichlin ML , Ball DE , Matthews BR . Disease severity and minimal clinically important differences in clinical outcome assessments for Alzheimer's disease clinical trials. Alzheimers Dement. 2019;5(1):354‐363. 10.1016/j.trci.2019.06.005 PMC6690415 31417957

[43] Marshall GA , Rentz DM , Frey MT , et al. Executive function and instrumental activities of daily living in mild cognitive impairment and Alzheimer's disease. Alzheimers Dement. 2011;7(3):300‐308. 21575871 10.1016/j.jalz.2010.04.005 PMC3096844

[44] Page MJ , Mckenzie JE , Bossuyt PM , et al. The PRISMA 2020 statement: an updated guideline for reporting systematic reviews. BMJ. 2021;372:n71. 33782057 10.1136/bmj.n71 PMC8005924

