# Supplementary material for: Assessing clinical progression measures in Alzheimer's disease trials: A systematic review and meta‐analysis
Source: Alzheimers Dement. 2024 Oct 22;20(12):8673–83. doi: 10.1002/alz.14314 (PMC11667530; doi:10.1002/alz.14314)
Supplement: Supplementary file 1 — Supporting Information [file ALZ-20-8673-s001.docx]

## **APPENDIX I**

**PRISMA Guideline Adherence:**

***Table S1: PRISMA Guideline from Page MJ, et al. The PRISMA 2020 statement: an updated guideline for reporting systematic reviews. BMJ 2021.***

| **Section and Topic** | **Item #** | **Checklist item** | **Location where item is reported** |
| --- | --- | --- | --- |
| **TITLE** | | |  |
| Title | 1 | Identify the report as a systematic review. | p 1 |
| **ABSTRACT** | | |  |
| Abstract | 2 | See the PRISMA 2020 for Abstracts checklist. | p 2 |
| **INTRODUCTION** | | |  |
| Rationale | 3 | Describe the rationale for the review in the context of existing knowledge. | pp 3-4 |
| Objectives | 4 | Provide an explicit statement of the objective(s) or question(s) the review addresses. | p 4 |
| **METHODS** | | |  |
| Eligibility criteria | 5 | Specify the inclusion and exclusion criteria for the review and how studies were grouped for the syntheses. | pp 6-7 |
| Information sources | 6 | Specify all databases, registers, websites, organisations, reference lists and other sources searched or consulted to identify studies. Specify the date when each source was last searched or consulted. | Appendix **I** pp 4-7 |
| Search strategy | 7 | Present the full search strategies for all databases, registers and websites, including any filters and limits used. | Appendix **I** pp 4-6 |
| Selection process | 8 | Specify the methods used to decide whether a study met the inclusion criteria of the review, including how many reviewers screened each record and each report retrieved, whether they worked independently, and if applicable, details of automation tools used in the process. | p 6  Appendix **I** pp 7-8 |
| Data collection process | 9 | Specify the methods used to collect data from reports, including how many reviewers collected data from each report, whether they worked independently, any processes for obtaining or confirming data from study investigators, and if applicable, details of automation tools used in the process. | pp 7-8 |
| Data items | 10a | List and define all outcomes for which data were sought. Specify whether all results that were compatible with each outcome domain in each study were sought (e.g. for all measures, time points, analyses), and if not, the methods used to decide which results to collect. | Appendix **I** pp 10-18  **Table 1** |
|  | 10b | List and define all other variables for which data were sought (e.g. participant and intervention characteristics, funding sources). Describe any assumptions made about any missing or unclear information. | Appendix **I** pp 10-18  **Table 2** |
| Study risk of bias assessment | 11 | Specify the methods used to assess risk of bias in the included studies, including details of the tool(s) used, how many reviewers assessed each study and whether they worked independently, and if applicable, details of automation tools used in the process. | Appendix **II** pp 22-24 |
| Effect measures | 12 | Specify for each outcome the effect measure(s) (e.g. risk ratio, mean difference) used in the synthesis or presentation of results. | p 7 |
| Synthesis methods | 13a | Describe the processes used to decide which studies were eligible for each synthesis (e.g. tabulating the study intervention characteristics and comparing against the planned groups for each synthesis (item #5)). | n/a |
|  | 13b | Describe any methods required to prepare the data for presentation or synthesis, such as handling of missing summary statistics, or data conversions. | pp 8-9 |
|  | 13c | Describe any methods used to tabulate or visually display results of individual studies and syntheses. | n/a |
|  | 13d | Describe any methods used to synthesize results and provide a rationale for the choice(s). If meta-analysis was performed, describe the model(s), method(s) to identify the presence and extent of statistical heterogeneity, and software package(s) used. | pp 8-10  Appendix **III** & **IV** |
|  | 13e | Describe any methods used to explore possible causes of heterogeneity among study results (e.g. subgroup analysis, meta-regression). | pp 8-9 |
|  | 13f | Describe any sensitivity analyses conducted to assess robustness of the synthesized results. | n/a |
| Reporting bias assessment | 14 | Describe any methods used to assess risk of bias due to missing results in a synthesis (arising from reporting biases). | n/a |
| Certainty assessment | 15 | Describe any methods used to assess certainty (or confidence) in the body of evidence for an outcome. | n/a |
| **RESULTS** | | |  |
| Study selection | 16a | Describe the results of the search and selection process, from the number of records identified in the search to the number of studies included in the review, ideally using a flow diagram. | **Figure 1** |
|  | 16b | Cite studies that might appear to meet the inclusion criteria, but which were excluded, and explain why they were excluded. | Appendix **I** pp 8-9 |
| Study characteristics | 17 | Cite each included study and present its characteristics. | Appendix **I** pp 10-18 |
| Risk of bias in studies | 18 | Present assessments of risk of bias for each included study. | Appendix **II**  **Figure S2** |
| Results of individual studies | 19 | For all outcomes, present, for each study: (a) summary statistics for each group (where appropriate) and (b) an effect estimate and its precision (e.g. confidence/credible interval), ideally using structured tables or plots. | Appendix **III**  **Table S5**  Appendix **IV**  **Figures S3-S14** |
| Results of syntheses | 20a | For each synthesis, briefly summarise the characteristics and risk of bias among contributing studies. | Appendix **II**  **Figure S2** |
|  | 20b | Present results of all statistical syntheses conducted. If meta-analysis was done, present for each the summary estimate and its precision (e.g. confidence/credible interval) and measures of statistical heterogeneity. If comparing groups, describe the direction of the effect. | Appendix **III**  Appendix **IV** |
|  | 20c | Present results of all investigations of possible causes of heterogeneity among study results. | n/a |
|  | 20d | Present results of all sensitivity analyses conducted to assess the robustness of the synthesized results. | n/a |
| Reporting biases | 21 | Present assessments of risk of bias due to missing results (arising from reporting biases) for each synthesis assessed. | n/a |
| Certainty of evidence | 22 | Present assessments of certainty (or confidence) in the body of evidence for each outcome assessed. | Appendix **III**  **Table S5** |
| **DISCUSSION** | | |  |
| Discussion | 23a | Provide a general interpretation of the results in the context of other evidence. | pp 10-15 |
|  | 23b | Discuss any limitations of the evidence included in the review. | pp 13-15 |
|  | 23c | Discuss any limitations of the review processes used. | pp 13-15 |
|  | 23d | Discuss implications of the results for practice, policy, and future research. | pp 3-4 ; 12-15 |
| **OTHER INFORMATION** | | |  |
| Registration and protocol | 24a | Provide registration information for the review, including register name and registration number, or state that the review was not registered. | n/a |
|  | 24b | Indicate where the review protocol can be accessed, or state that a protocol was not prepared. | n/a |
|  | 24c | Describe and explain any amendments to information provided at registration or in the protocol. | n/a |
| Support | 25 | Describe sources of financial or non-financial support for the review, and the role of the funders or sponsors in the review. | p 19 |
| Competing interests | 26 | Declare any competing interests of review authors. | p 19  ICMJE disclosure form |
| Availability of data, code and other materials | 27 | Report which of the following are publicly available and where they can be found: template data collection forms; data extracted from included studies; data used for all analyses; analytic code; any other materials used in the review. | GitHub link  available  on request |

For more information, visit: <http://www.prisma-statement.org/>

## Database search terms

**Alzheimer’s disease Clinical trials with Patient Amyloid Status documented (By PET or CSF)**

**Ovid MEDLINE(R) <1946 to April 8^th^ 2022>**

1 alzheimer*.ti. or exp *alzheimer disease/

2 (cerebrospinal fluid or CSF).ti.

3 Positron-Emission Tomography/

4 exp *amyloid/

5 2 or 3 or 4

6 1 and 5

7 (prognos* or progression).tw.

8 exp prognosis/

9 exp disease progression/

10 exp epidemiologic studies/

11 7 or 8 or 9 or 10

12 6 and 11

13 controlled trial.tw.

14 (cohort or prospective or retrospective or follow-up or longitudinal).tw.

15 cross-sectional.tw. or cross-sectional/

16 Pragmatic Clinical Trial/ or Clinical Trial, Phase II/ or Controlled Clinical Trial/ or Clinical Trial, Phase I/ or Randomized Controlled Trial/ or Clinical Trial/ or Clinical Trial, Phase III/ or Clinical Trial, Phase IV/

17 (drug or therapeutic or medication).mp. [mp=title, abstract, original title, name of substance word, subject heading word, floating sub-heading word, keyword heading word, organism supplementary concept word, protocol supplementary concept word, rare disease supplementary concept word, unique identifier, synonyms]

18 13 or 14 or 15 or 16 or 17

19 18 not 15

20 12 and 19

21 (mouse or mice or murine or rat or rats or animal or rodent or monkey or primate or drosophila).ti.

22 20 not 21

23 limit 22 to yr="2005 -Current"

24 limit 23 to english language

**Total retrieved= 1687**

**Alzheimer’s disease Clinical trials with Patient Amyloid Status documented (By PET or CSF)- version 2**

**Ovid Embase <1974 to April 8^th^ 2022>**

1 alzheimer*.ti. or exp *alzheimer disease/

2 (cerebrospinal fluid or CSF).ti.

3 Positron-Emission Tomography/

4 exp *amyloid/

5 2 or 3 or 4

6 1 and 5

7 (prognos* or progression).tw.

8 exp prognosis/

9 exp disease progression/

10 exp epidemiologic studies/

11 7 or 8 or 9 or 10

12 6 and 11

13 controlled trial.tw.

14 (cohort or prospective or retrospective or follow-up or longitudinal).tw.

15 cross-sectional.tw. or cross-sectional/

16 Pragmatic Clinical Trial/ or Clinical Trial, Phase II/ or Controlled Clinical Trial/ or Clinical Trial, Phase I/ or Randomized Controlled Trial/ or Clinical Trial/ or Clinical Trial, Phase III/ or Clinical Trial, Phase IV/

17 (drug or therapeutic or medication).mp. [mp=title, abstract, original title, name of substance word, subject heading word, floating sub-heading word, keyword heading word, organism supplementary concept word, protocol supplementary concept word, rare disease supplementary concept word, unique identifier, synonyms]

18 13 or 14 or 15 or 16 or 17

19 18 not 15

20 12 and 19

21 (mouse or mice or murine or rat or rats or animal or rodent or monkey or primate or drosophila).ti.

22 20 not 21

23 limit 22 to yr="2005 -Current"

24 limit 23 to english language

**Total retrieved= 1495**

**Deduplicated= 2744**

### Search of Trial Registers

A first search was undertaken of the National Institute of Health trial registry at ClinicalTrials.gov in April 2022. This was with the parameters as follows:

1. “Alzheimer Disease” (a subject heading in the registry)
2. Completed trials
3. With results
4. Interventional
5. Adult (18-64)
6. Adult (65+)

This yielded 321 records which were then screened and 298 were excluded leaving 23 records for further review. Of these records 13 were eligible for inclusion in the review.

A second search of the same registry was performed in May 2022. This time additional search parameters were chosen to widen the search to clinical trials that had published results but had not been completed trials. For example, this could be due to early termination or the study sponsor, often a drug company, withdrawing as sponsor or for other reasons ending the trial. The parameters chosen are as follows:

1. “Alzheimer Disease” (a subject heading in the registry)
2. With results
3. Interventional
4. Adult (18-64)
5. Adult (65+)

This search yielded 405 records. A total of 37 records warranted full text review after screening. Of these records 9 trials were eligible for inclusion giving a total 25 studies. An additional extensive search of other available databases was made using the resources listed above. This did not identify any additional studies for inclusion. There was significant overlap in the trial registries. Studies found in the 2 searches of ClinicalTrials.gov were often found in the other registries, particularly the ICTRP WHO. A search of compiled pharmaceutical company press releases via <https://pharmaintelligence.infomra.com> did not locate any eligible studies.

1. Research Question

A search of the International Prospective Register of Systematic Reviews (PROSPERO) and The Cochrane Library revealed that no similar review had been undertaken.

The research question was formulated ahead of the search strategy. The Population, Intervention, Control, Outcomes and Study Design principle (PICOS) informed this:

***Population:*** Males and Females over 18 years of age with a diagnosis of AD, MCI due to AD pathology or Prodromal AD in whom AD pathology has been established by the presence of elevated levels of amyloid protein on Amyloid PET or via CSF analysis prior to inclusion in a clinical trial. Only placebo groups were used in the review.

***Intervention:*** Studies investigating therapeutic agents for the treatment of Alzheimer’s disease whether this be in established AD or Prodromal AD or MCI due to AD pathology.

***Control:*** Not applicable – the placebo groups in the therapeutic trials formed the target population for this review.

***Outcomes:*** The assessment of progression in Alzheimer’s disease via novel clinical progression measures/models or established clinical measures (e.g. ADAS-cog, MMSE, CDR-SB).

***Study design:*** Randomised Controlled Trials (RCTs) were exclusively sought.

This process refined the research question underpinning the systematic review;

*To analyse the methods of measuring clinical progression in Randomised Controlled Trials in Alzheimer’s Disease, specifically using placebo arm patients with known positive amyloid status prior to trial entry.*

## Inclusion and Exclusion Criteria

Studies were included if they met the defined criteria as follows:

- RCT in design and trialling a therapeutic intervention.
- RCTs that used clinical outcome/progression measures.
- Participant inclusion criteria stipulated that only Aβ+ subjects/subjects with elevated Aβ were included in the trial as determined by either Amyloid PET or CSF analysis.
- Subjects had a diagnosis of AD or were Cognitively Normal (CN) but with evidence of Aβ deposition.
- The RCT must have a placebo group and have reported data on clinical outcome/progression measures for the placebo group. In circumstances where the RCT did not have a published report in a peer reviewed journal it could be included if data was reported on a recognised forum such as ClinicalTrials.gov.

Studies were excluded if they met the defined criteria as follows:

- Observational, case study or reports, case control or cohort study in design
- RCTs which report elevated tau levels but in which there is no account of Aβ+
- RCTs which included patients with other forms of dementia/pathology e.g. suspected cerebral vascular disease
- RCTs which trialled an intervention for symptoms/difficulties arising other than cognitive decline
- RCTs which trialled an intervention aimed at caregivers e.g. education intervention
- Insufficient data reported e.g. no data on change in clinical measures from baseline to endpoint or idiosyncratic and sparse clinical measures used.

No limits were placed on the number of trial participants, purported severity of AD, length of the trial, class of therapeutic agent, the main outcome of the trial, and the number of clinical outcome/progression measures used.

## Resources for search

The following resources were used for the study search; MEDLINE (Ovid interface), Embase (OVID interface), PubMed, Google Scholar, National Institute of Health ClinicalTrials.gov, AIBL list of publications, International Clinical Trials Registry Platform (ICTRP WHO), International Standard Randomised Controlled Trial Number registry (ISRCTN), European Union Drug Regulating Authorities Clinical Trials Database (EudraCT) and Researchregistry.com.

### Scoping search

An initial scoping electronic search was devised and refined, *Appendix I*. The purpose was to define and optimise search terms, ensure no similar and previous review had been undertaken (prior PROSPERO and Cochrane library searches had suggested not) and to establish whether there was enough literature to proceed. On this later point, and on review of titles and abstracts of the 2,744 records found in the scoping search, 117 studies were identified for full text review. Of these only 3 records fulfilled criteria for inclusion. The scoping search was successful in helping to identify relevant keywords and Medical Subject Headings (MESH terms) as well as allowing identification of related reviews which had published their search strategies e.g. ^1^. Additionally, via scoping it became clear that a significant proportion of trials that met the inclusion criteria would potentially be subject to publication bias as they had statistically non-significant outcomes. This, in part, would explain the low rate of studies found on database searching.

### Table of Studies identified through database and trial registry searches & PRISMA checklist

*Table S2 : Summary of identified studies detailing; active intervention, outcome measures used, summary notes on main findings and additional details.*

|  | Author /  Company  &  Year | Trial Number(s) | Study Name  Active Intervention | Amyloid status known | Number of patients | Endpoint | ADAS  -COG | MMSE | CDR  -SB | FAQ | Statistical reporting method | Main finding | Other progression measures used and notes |
| --- | --- | --- | --- | --- | --- | --- | --- | --- | --- | --- | --- | --- | --- |
| 1 | **Astrazeneca**  **&**  **National Institute on ageing (NIA)**  **2018**  **Mullins et al.**  **2019** | NCT01255163 | Exendin-4 | Yes  CSF | 27  13 Int.  14 Plb. | 18 mnths | Y | Y | Y | - | Mean score & SD | Study terminated after AstraZeneca withdrew support for study. | CDR-GS  ADCS |
| 2 | **Eli Lilly and Company**  **2018** | NCT01561430 | LY2886721 | Yes  PET | 70  50 Int.  20 Plb. | 26 wks | Y | Y | Y | - | Least square means & SE | Study terminated due to abnormal liver function tests in some participants | - |
| 3 | **Biogen**  **2021** | NCT02477800 | Aducanumab  ENGAGE | Yes  PET | 1647  1102 Int.  545 Plb. | 78 wks | Y | Y | Y | - | Mean Change & SE | Study discontinued on Futility analysis | ADCS-ADL-MCI |
| 4 | **Biogen**  **2021** | NCT02484547 | Aducanumab  EMERGE | Yes  PET | 1638  1090 Int.  548 Plb. | 78 wks | Y | Y | Y | - | Mean Change & SE | Study discontinued on Futility analysis | ADCS-ADL-MCI |
| 5  * | **Sperling et al.**  **2021**  **Janssen R&D LLC** | NCT02569398 | Atabecestat  EARLY | Yes  PET and/or CSF | 557  372 Int.  185 Plb. | 104 wks | - | - | Y | - | Mean change & SD/  Z-score & SD | Atabecestat elevated liver enzymes leading to change in benefit-risk profile  Study terminated | CDR-SB -not completed for participants  PACC (comp.)  CFI  ADCS-ADLPI  RBANS  NABDLTS |
| 6 | **Hoffman-La Roche**  **2020** | NCT02670083 | Crenezumab  CREAD | Yes  PET and/or CSF | 813  404 Int.  409 Plb. | 105 wks | Y | Y | Y | - | Least square means & SE | Crenezumab did not meet primary endpoint of change in CDR-SB  Study terminated at interim analysis | CDR-GS  ADCS-ADL- no placebo data  ADCS-iADL  NPI  QoL-AD  ZCI-AD  EQ-5D |
| 7 | **Eli Lilly and Company**  **2021** | NCT02791191 | LY3202626  NAVIGATE-AD | Yes  PET | 316  183 Int.  133 Plb. | 52 wks | Y | - | - | - | Least square means & SE | LY3202626 did not affect cognitive decline | ADCS-iADL  iADRS |
| 8 | **Hoffman-La Roche**  **2020** | NCT03114657 | Crenezumab  CREAD 2 | Yes  PET and/or CSF | 806  407 Int.  399 Plb. | 77 wks | Y | Y | Y | Y | Least square means & SE | Crenezumab did not affect cognitive decline  Study terminated at interim analysis | CDR-GS  ADCS-ADL  ADCS-iADL  FAQ  NPI  QoL-AD  ZCI-AD  EQ-5D |
| 9 | **Novartis**  **2021** | NCT03131453 | CNP520 | Yes  PET and/or CSF | 1145  689 Int.  456 Plb. | 648 days | - | - | Y | - | Mean change & SD | CNP520 did not affect cognitive decline (safety issues with drug) | APCC  RBANS  ECOG |
| 10 | **Genentech inc.**  **2022** | NCT03289143 | Semorinemab | Yes  PET and/or CSF | 457  322 Int.  135 Plb. | 73 wks | Y | - | Y | - | Mean change & SE | Semorinemab did not affect cognitive decline | RBANS  iADL  ADCS-ADL |
| 11 | **Eisai Inc.**  **&**  **Biogen**  **2021** | NCT02956486 | MissionAD1  MIssionAD2  (Pooled)  Elenbecestat | Yes  PET  and/or  CSF | 2131  1053 Int.  1078 Plb. | 104 wks | Y | Y | Y | Y | Least square means & SE  Least Square Mean difference w/p-value | Elenbecestat did not affect cognitive decline | ADCOMS  FAQ  Core phase leading to not undertaken extension phase of study |
| 12 | **Wessels et al.**  **2019**  **& Astrazeneca/Eli Lily** | NCT02245737  NCT02783573 | AMARANTH  DAYBREAK-ALZ  Lanabecestat | Yes  PET and/or CSF | AMARANTH  2218  1478 Int.  740 Plb.  DAYBREAK-ALZ  1722  1160 Int.  562 Plb. | 104 wks &  78wks | Y | Y | Y | Y | Least square means & SE  Least Square Mean difference w/p-value | Lanabecestat did not affect cognitive decline | ADCS-iADL  FAQ  iADRS  NPI |
| 13 | **Egan et al.**  **2019**  **&**  **Merck Sharp & Dohme LLC.** | NCT01953601 | Verubecestat  APECS | Yes  PET | 1454  969 Int.  485 Plb. | 104 wks | Y | Y | Y | - | Least square means & CI | Verubecestat did not affect cognitive decline- on some measures worsened it | NPI  ADAS-Cog 13&11 |
| 14 | **Coric et al.**  **2015**  **& Bristol- Meyers Squibb** | NCT00890890 | Avagcestat | Yes  CSF | 137  59 Int.  78 Plb. | 104 wks | Y | Y | Y | - | Mean change & SE | Avagcestat did not affect cognitive decline | ADCS ADL-MCI |
| 15 | **Honig et al.**  **2018**  **& Eli Lily and Company** | NCT01900665 | Solanezumab  EXPEDITION 3 | Yes  PET and/or  CSF | 1822  914 Int.  908 Plb. | 80 wks | Y | Y | Y | Y | Least square means & SE | Solanezumab did not affect cognitive decline | FAQ  ADCS-ADL  ADCS-iADL  iADRS  NPI  QoL-AD |
| 16 | **Swanson et al.**  **2021**  **& Eisai** | NCT01767311 | Lecanemab | Yes  PET  And/or  CSF | 854  609 Int.  245 Plb. | 18 mnths | Y | Y | Y | - | Least square means & SE | Slower clinical decline on Lecanemab but missing primary target of relative 80% less/slower decline on ADCOMS | ADCOMS  (4 ADAS-COG SUBSCALES/2MMSE ITEMS/ALL 6 CDR-SB) |
| 17  ** | **Potter et al.**  **2021** | NCT01409915 | Sargramostim | Yes  PET | 40  20 Int.  20 Plb. | 90 days | Y | Y | Y | - | Mean change & SD | MMSE increased compared to baseline and Placebo group (p= sig) | ADCS-ADL  Trailmaking Test-A |
| 18 | **van Dyck et al.**  **2016**  **Janssen**  **&**  **Pfizer** | NCT01227564 | ACC-001 | Yes  PET | 63  42 Int.  21 Plb. | 104 wks | Y | Y | Y | Y | Least square means  &  95% CI | No change in amyloid burden | FAQ  Primary outcome measure was Amyloid SUVR |
| 19 | **Klein et al.**  **2019**  **&**  **Hoffman-La Roche** | NCT01224106  NCT02051608 | Gantenerumab  Scarlet Road  Marguerite Road | Yes  PET | SCARLET ROAD  797  531 Int.  266 Plb. | 104 wks | Y | Y | Y | Y | Mean change & SD | Gantenerumab reduces amyloid no clinical improvement | FAQ  NPI  Primary outcome measure was Amyloid SUVR  **missing Marguerite Road data** |
| 20*** | **Cummings et al.**  **2016** | NCT01782742 | Bexarotene  BEAT-AD | Yes  PET | 20  16 Int.  4 Plb. | 28 days | Y | Y | Y | - | Mean Change & 95 % CI | Bexarotene reduces amyloid no clinical improvement | NPI  ADCS-ADL  Primary outcome was Amyloid SUVR |
| 21 | **Van Dyck et al.**  **2019** | NCT02167256 | AZD0530 | Yes  PET | 159  80 Int.  79 Plb. | 52 wks | Y | Y | Y | - | Mean change & SE | No effect of AZD0530 on metabolic or cognitive decline | NPI  ADCS-ADL  Primary outcome was Cerebral metabolic decline by FDG-PET |
| 22 | **Frolich et al.**  **2019**  **Boehringer Ingelheim** | NCT02240693 | BI409306 Phosphodiesterase inhibitor | Yes  PET and/or CSF | 128  85 Int.  43 Plb. | 12 wks | Y | - | Y | - | Least square means & SE |  | ADCS-MCI-ADL |
| 23 | **Prins et al.**  **2021**  **&**  **EIP Pharma Inc.** | NCT03402659 | Neflamapimod  REVERSE SD | Yes  CSF | 161  78 Int.  83 Plb. | 24 wks | - | Y | Y | - | Mean change & SE | No sig. differences between treatment groups | HVLT-R (primary outcome measure)  WMS Immediate and delayed recall |
| 24 | **Mintun et al.**  **2021**  **&**  **Eli Lilly and company** | NCT03367403 | Donanemab  TRAILBLAZER-ALZ | Yes  PET | 257  131 Int.  126 Plb. | 76 wks | Y | Y | Y | - | Least square means & SE | Small difference in favour of Donanemab vs placebo | iADRS (primary measure)  ADCS-iADL |
| 25 | Wang et al.  &  Cassava sciences inc.  2021 | NCT04079803 | Simufilam | Yes  CSF | 64  42 Int.  22 Plb. | 28 days | - | - | - | - | Mean change & SD | Less errors on drug vs placebo but odd test choices and short-28 days | *Unpublished  Paired associates learning  Spatial working memory  ***excluded from statistical analysis as outcome measures only used in this study and 28 days*** |

* Early termination of study led to CDR-SB not being completed. Other Measures used in 2 or fewer studies. Data not included in analysis.

** Trial only 90 days in duration. Data not included in analysis.

*** Trial only 28 days in duration. Data not included in analysis.

### Diagnostic criteria comparison

*Table S3:* *Summary comparison of NINCDS/ADRDA (McKhann et al., 1984), NIA-AA (Jack et al., 2011) and IWG (Dubois et al., 2014) diagnostic criteria.*

| **NINCDS/ADRDA (1984)** | **Notes** | **NIA-AA (2011)** | **Notes** | **IWG (2014)** | **Notes** |
| --- | --- | --- | --- | --- | --- |
| **Possible AD** | Supported by;  -a dementia syndrome with variation in onset, clinical course and presentation  -a single gradually progressive severe cognitive deficit in the absence of another cause | **Preclinical AD** | Evidence of cerebral amyloid +/- neuronal injury and subtle cognitive change | **Asymptomatic at risk with AD pathology** | Pathophysiological marker with normal cognition |
| **Probable AD** | Supported by;  -Impaired ADLs  -Deficits in 2 or more areas of cognition including memory  -Progressive worsening  -Family history  -evidence of cerebral atrophy on CT with progression | **MCI due to AD** | **High/intermediate/possible and unlikely** **due to AD** sub-stratification based on presence or absence of amyloid and tau biomarkers | **Presymptomatic AD** | Autosomal dominant AD mutation with normal cognition |
| **Definite AD** | Supported by;  -clinical criteria for probable AD met  -histopathologic evidence obtained from biopsy or autopsy | **Dementia caused by AD** | Sub-stratification based on certainty levels e.g. **Probable**- dementia + evidence of AD pathological process (amyloid and tau) **Intermediate** (amyloid or tau present) | **Prodromal AD** | Episodic memory impairment and one biomarker e.g. amyloid imaging or CSF |
|  |  |  |  | **AD Dementia** | Episodic memory (or atypical AD phenotype- e.g. Posterior cortical atrophy) present with impaired ADLs and at least one biomarker e.g. CSF changes/positive amyloid imaging/medial temporal lobe atrophy on MRI |

## **Appendix II**

## Risk of Bias analysis

In line with best practice, as set out in Cochrane guidelines ^2^, Risk of Bias in the included RCTs was assessed using the revised tool to assess risk of bias in randomised trials (RoB 2) ^3^.

Bias refers to systematic error which causes a deviation from the truth in results or inferences ^2^. The RoB 2 tool assesses five main domains where bias may occur and includes bias because of the randomisation process, deviations from intended interventions, missing outcome data, measurement of the outcome and the selection of the reported result.

As is emphasised in the guidance around the use of the RoB 2 tool, despite the systematic approach, the process still involves making judgments about studies based on imperfect information ^3^. To minimise this imperfection as much as possible a number of sources were sought for each evaluated RCT. Main sources included study protocols and statistical analysis plans where these were available. In the 15 RCTs which had a published journal article this served as a further significant source. Most studies that did not have a published journal article did have both a study protocol and statistical analysis plan available in the trial registry ClinicalTrials.gov.

In **Domain 1**, the risk of bias arising from the **randomisation process**, most studies (eighteen of twenty-five) had robust randomisation procedures. Of the 7 studies which were classified as having some concerns in this area this was largely due to no detail on randomisation being provided other than a methods statement that the study was randomised. There were two studies, ^4,5^, in which baseline differences between intervention groups possibly suggested a problem with the randomisation process.

In **Domain 2**, the risk of bias due to **deviations from the intended interventions**, most studies (eighteen of twenty-five) were judged to have a low risk of bias. Six studies were judged to have a high risk. The main reason for this outcome via the RoB 2 algorithm was a lack of information about how deviations from the intervention, such as non-compliance, were to be handled in the analysis ^6,7^. In one instance there was an explicit statement that missing data, such as from participants who deviated from intervention, would not be imputed in the analysis^8^.

In **Domain 3**, the risk of bias due to **missing outcome data**, all studies were judged to be of low risk. A modifying factor which may have led to this judgement is that in the large majority of included RCTs (twenty-two of twenty-five) there was no reported significant effect from the active intervention. Missing data was common in the studies but it often stemmed from trials being terminated earlier than planned due to interim futility analysis ^9^ or concerns around adverse events ^10^. In these cases, it was not felt that the missingness was related to the true outcome (worsening cognition). Missingness in the circumstance of early termination affected intervention and placebo arms equally and is reflected in the observation that there were not significant differences in the proportions of missing data across intervention groups and placebo. That is to say that the missingness was not related to patients with worsening levels of impairment dropping out of the trial. There were studies which had outcome data for all or nearly all participants randomised although these tended to have small numbers of subjects our were relatively short in duration ^11,12^.

In **Domain 4**, the risk of bias due to **measurement of the outcome**, twenty-one of twenty-five studies were judged to be of low risk. The 4 remaining studies were all judged to be of high risk due to the use of outcome measures that could not with any confidence be said to be sensitive at picking up meaningful clinical change in the short duration of these trials. Two of the trials were of 28 days’ duration ^4,8^.

In the **final domain**, the risk of bias due to **selection of the reported result**, sixteen of the studies were judged to be low risk and 9 had some concerns. In most of these cases this was due to the absence of a pre-specified analysis plan which impaired the ability to assess how results were analysed and presented ^6,13^.

Overall, 9 of the studies were judged to be of low risk of bias with a further 8 judged as having some concerns leaving 8 as having high risk of bias. Of note is the relatively strong performance of studies in randomisation and in accounting for missing outcome data which is particularly relevant to the analysis of the outcome measures performance in the placebo groups.

*FIGURE S1: Visual summary of Risk of Bias domains for included studies.*


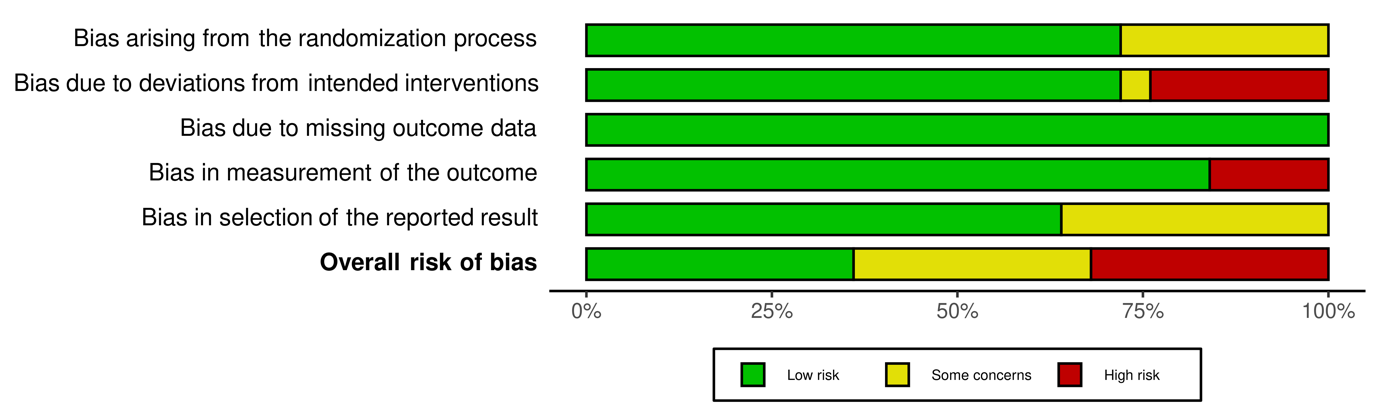


***FIGURE S2: Risk of Bias assessment of included studies using Cochrane Risk of Bias version 2 assessment tool***


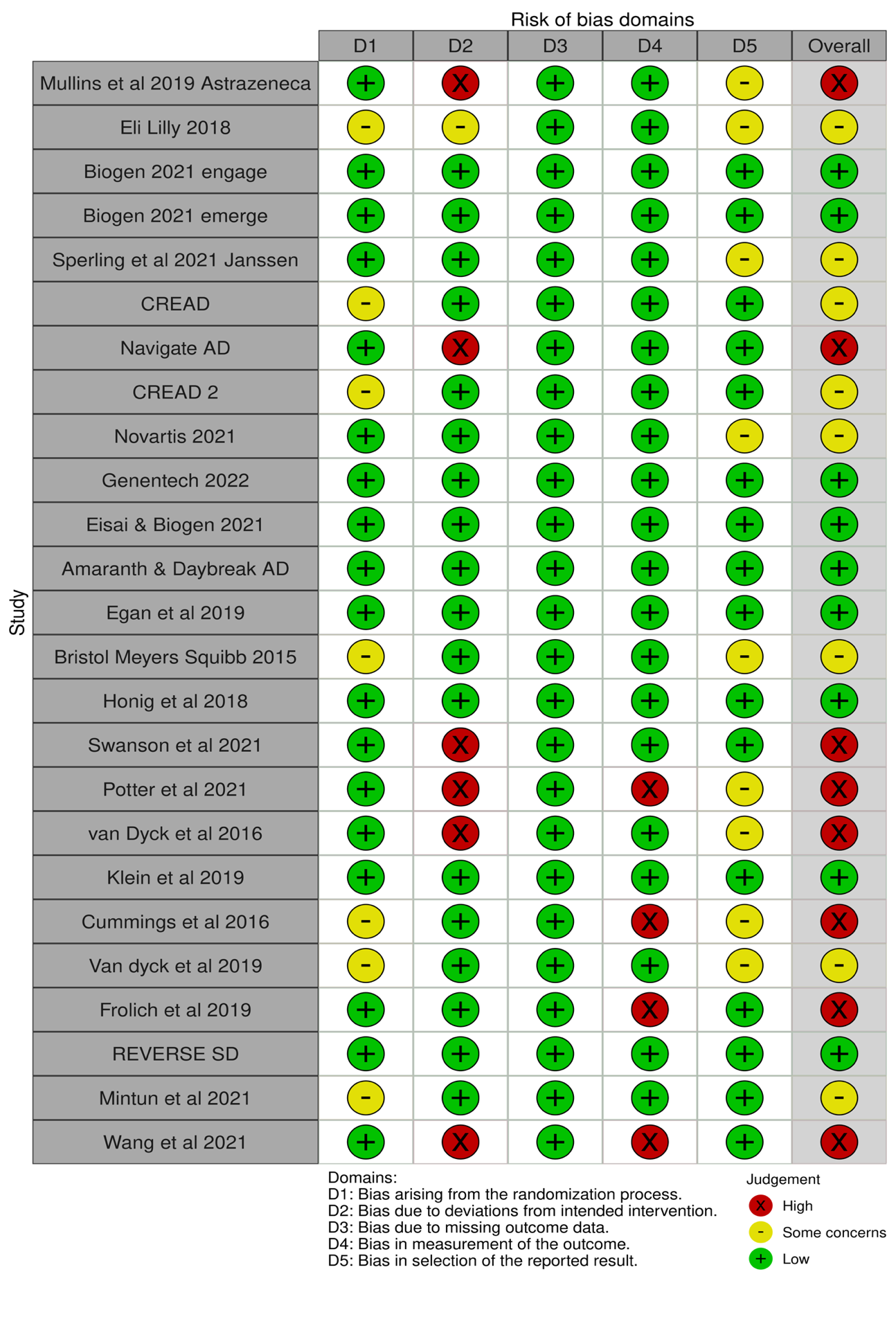


## **Appendix III**

*Table S4: Covariates used for meta regression for each progression measure.*

| *Test* | *Covariates available* |
| --- | --- |
| *ADCS-ADL-MCI* | *Age + percentage of females* |
| *ADCS-iADL* | *Age + percentage of females + disease stage* |
| *ADCS-ADL* | *Age + percentage of females + disease stage* |
| *FAQ* | *Age + percentage of females + disease stage* |
| *QOL-AD* | *disease stage* |
| *ADAS-Cog-11* | *Age + percentage of females + disease stage* |
| *ADAS-Cog-13* | *Age + percentage of females + disease stage* |
| *ADAS-Cog-14* | *disease stage* |
| *MMSE* | *Age + percentage of females + disease stage* |
| *CDRSB* | *Age + percentage of females + disease stage* |
| *iADRS* | *Age + percentage of females + disease stage* |
| *NPI* | *Age + percentage of females + disease stage* |

*Table S5: RESULTS OF THE META-ANALYSIS AS WELL AS META REGRESSION FOR EACH PROGRESSION MEASURE. *WMC=WEIGHTED MEAN CHANGE. P-VALUES CALCULATED FROM Z-SCORES.*

| *Test* | *raw *WMC* | *raw 95 %CI* | *raw P-value* | *Covaried WMC* | *Covaried 95 %CI* | *Covaried P-value* |
| --- | --- | --- | --- | --- | --- | --- |
| *ADCS_ADL_MCI* | *0.010* | *0.008-0.012* | *5.73E-28* | *0.012* | *0.010-0.014* | *5.69E-24* |
| *ADCS-iADL* | *0.017* | *0.014-0.020* | *9.85E-30* | *0.017* | *0.012-0.022* | *4.40E-10* |
| *ADCS-ADL* | *0.017* | *0.016-0.018* | *1.68E-163* | *0.018* | *0.015-0.020* | *2.52E-37* |
| *FAQ* | *0.017* | *0.016-0.018* | *1.68E-163* | *0.021* | *0.019-0.023* | *2.32E-94* |
| *QOL-AD* | *0.004* | *0.001-0.006* | *4.96E-04* | *0.004* | *0.0024-0.006* | *9.95E-06* |
| *ADAS cog11* | *0.011* | *0.007-0.014* | *1.37E-10* | *0.010* | *0.008-0.013* | *8.66E-17* |
| *ADAS cog13* | *0.010* | *0.008-0.013* | *1.06E-23* | *0.011* | *0.009-0.012* | *9.60E-28* |
| *ADAS cog14* | *0.009* | *0.006-0.0123* | *7.02E-09* | *0.009* | *0.009-0.010* | *6.29E-105* |
| *MMSE* | *0.009* | *0.006-0.0123* | *7.02E-09* | *0.017* | *0.016-0.019* | *5.20E-84* |
| *CDRSB* | *0.014* | *0.011-0.017* | *4.85E-22* | *0.015* | *0.012-0.017* | *1.02E-24* |
| *iADRS* | *0.014* | *0.011-0.016* | *2.25E-35* | *0.014* | *0.012-0.016* | *3.24E-32* |
| *NPI* | *0.001* | *0.001-0.002* | *1.79E-03* | *0.002* | *0.001-0.003* | *4.63E-04* |

**Appendix IV**

Meta-analysis of each measure of progression Figures S3-S14 show individual forest plots for the meta‑analysis of each measure of progression grouped by four metagroups: Cognitive, Functional, Composite, and Neuropsychiatric. With number of samples and studies indicated in each caption.

*FIGURE S3: Meta-analysis of ADS-ADL-MCI tool which comprises primarily functional measures. N=1752 across 5 studies.*


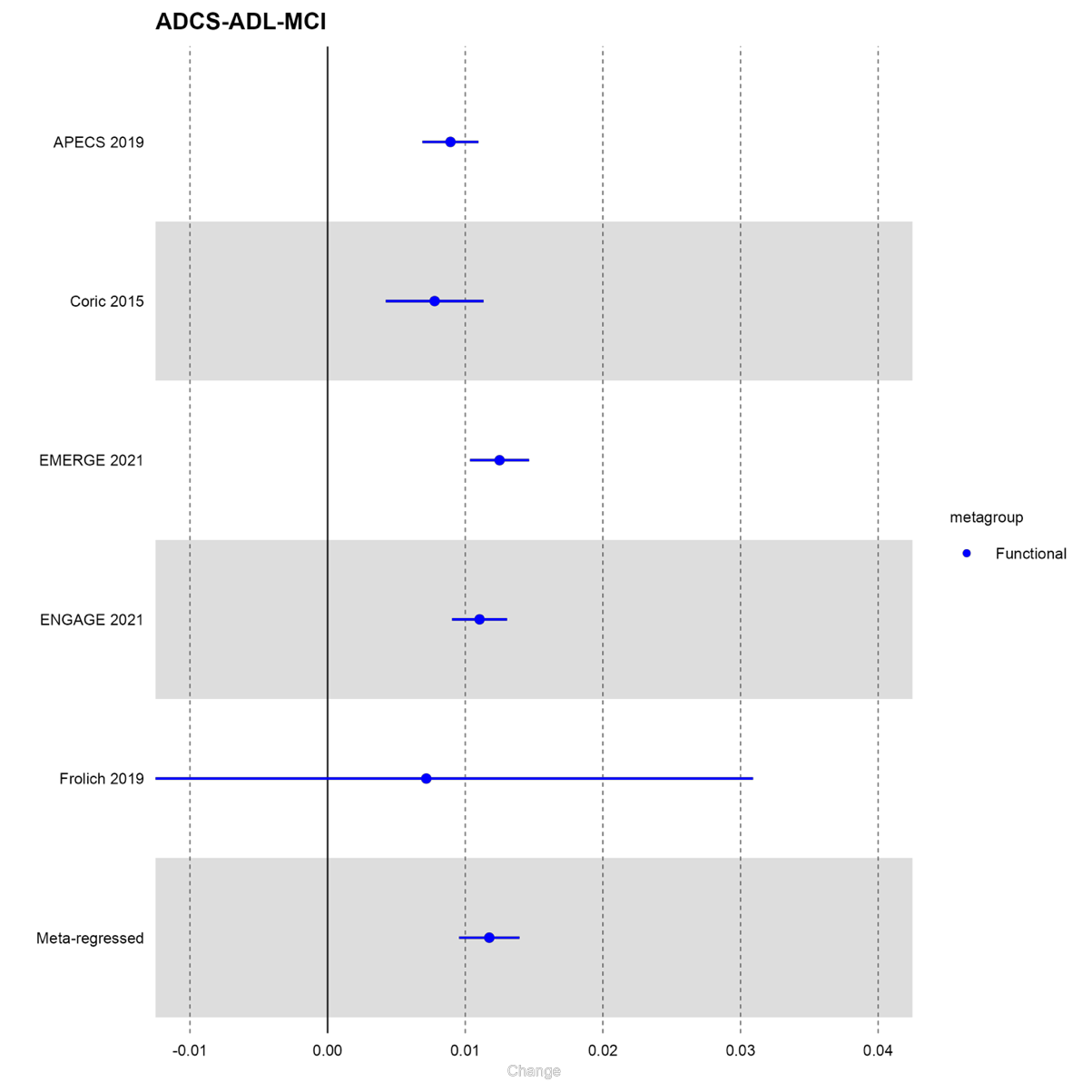


*FIGURE S4: Meta-analysis of ADS-iADL tool which comprises primarily functional measures. N=3441 across 7 studies.*


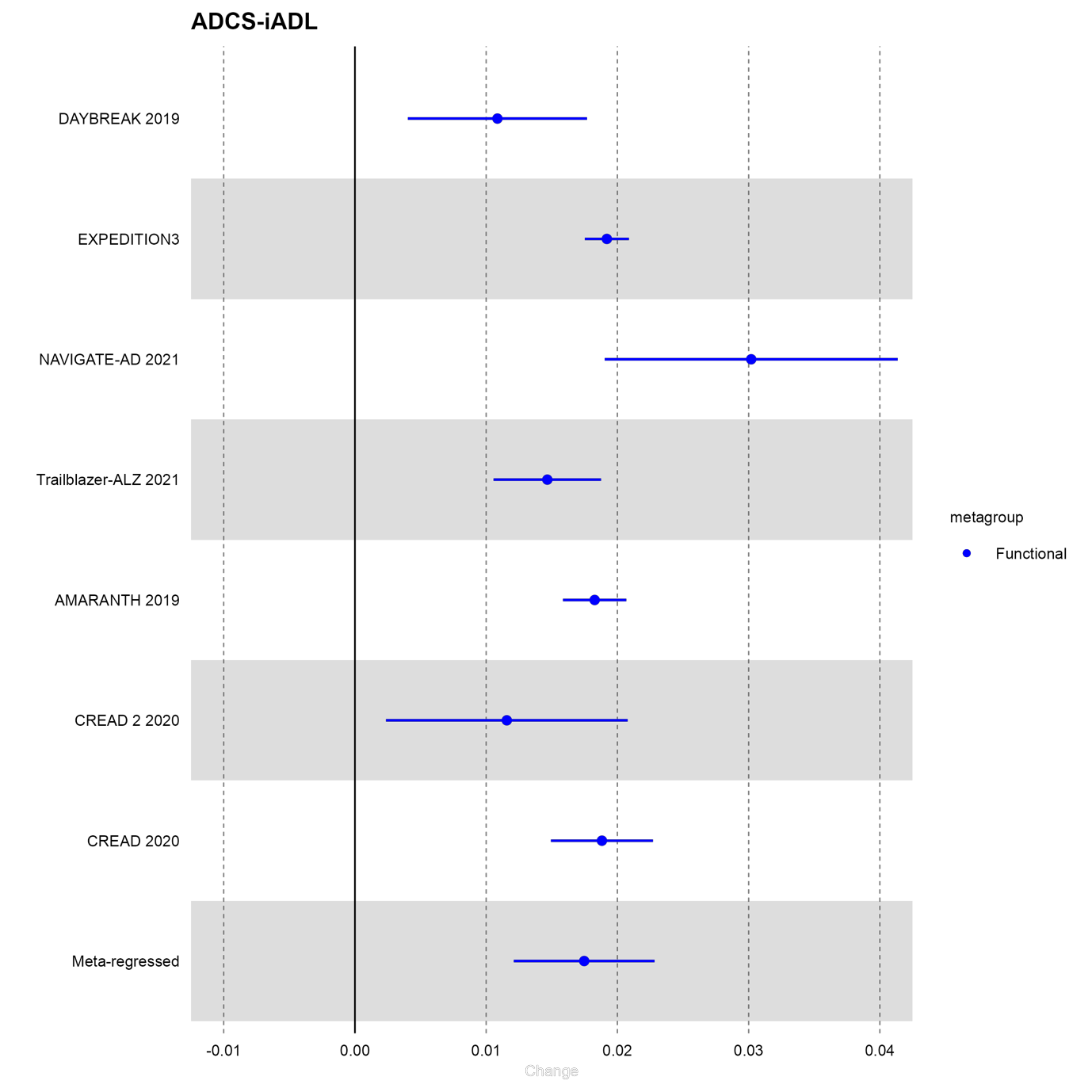


*FIGURE S5: Meta-analysis of ADS-ADL tool which comprises primarily functional measures. N=2109 across 6 studies.*


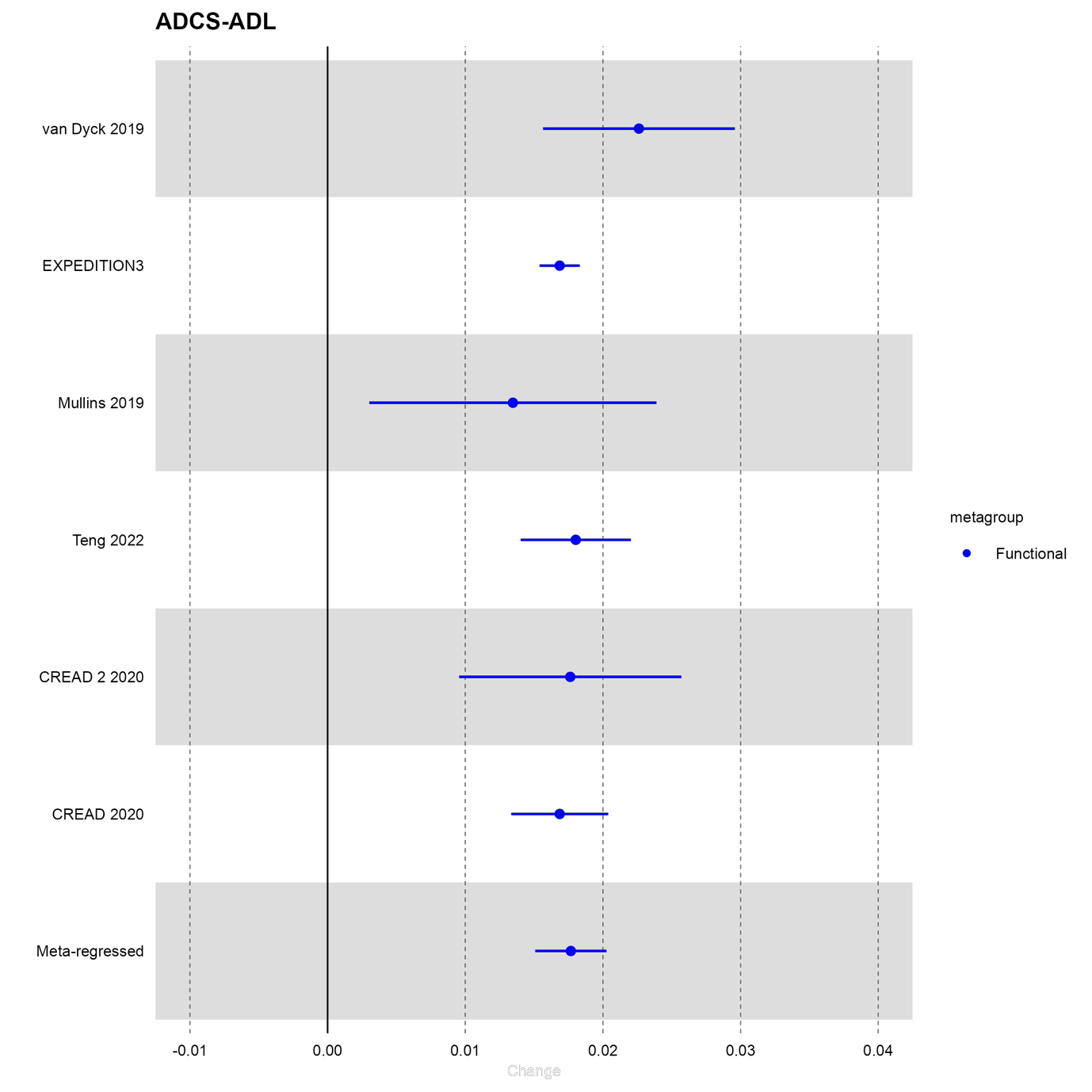


*FIGURE S6: Meta-analysis of FAQ tool which comprises primarily functional measures. N=4168 across 7 studies.*


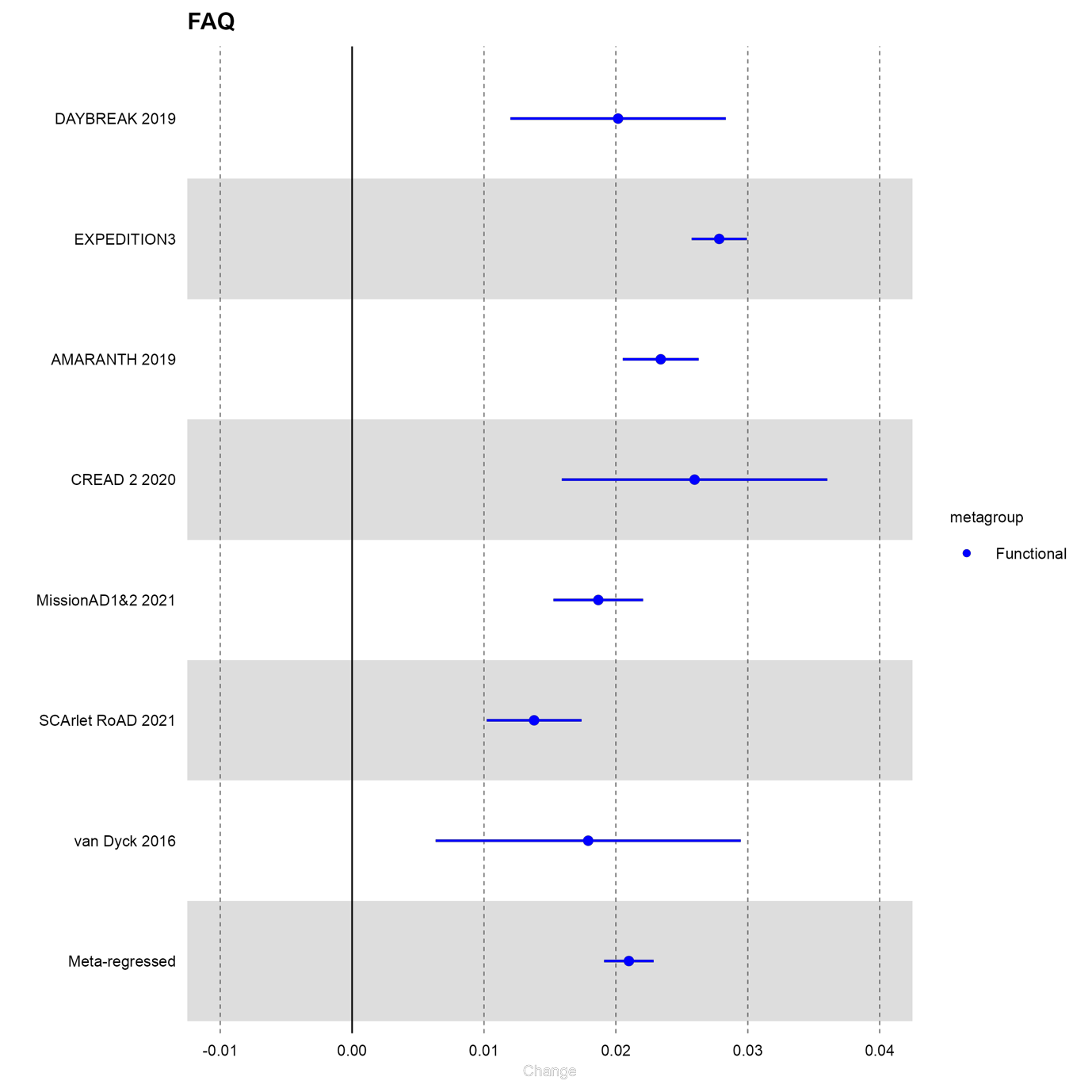


*FIGURE S7: Meta-analysis of QoL-AD tool which comprises primarily functional measures. N=1880 across 3 studies.*


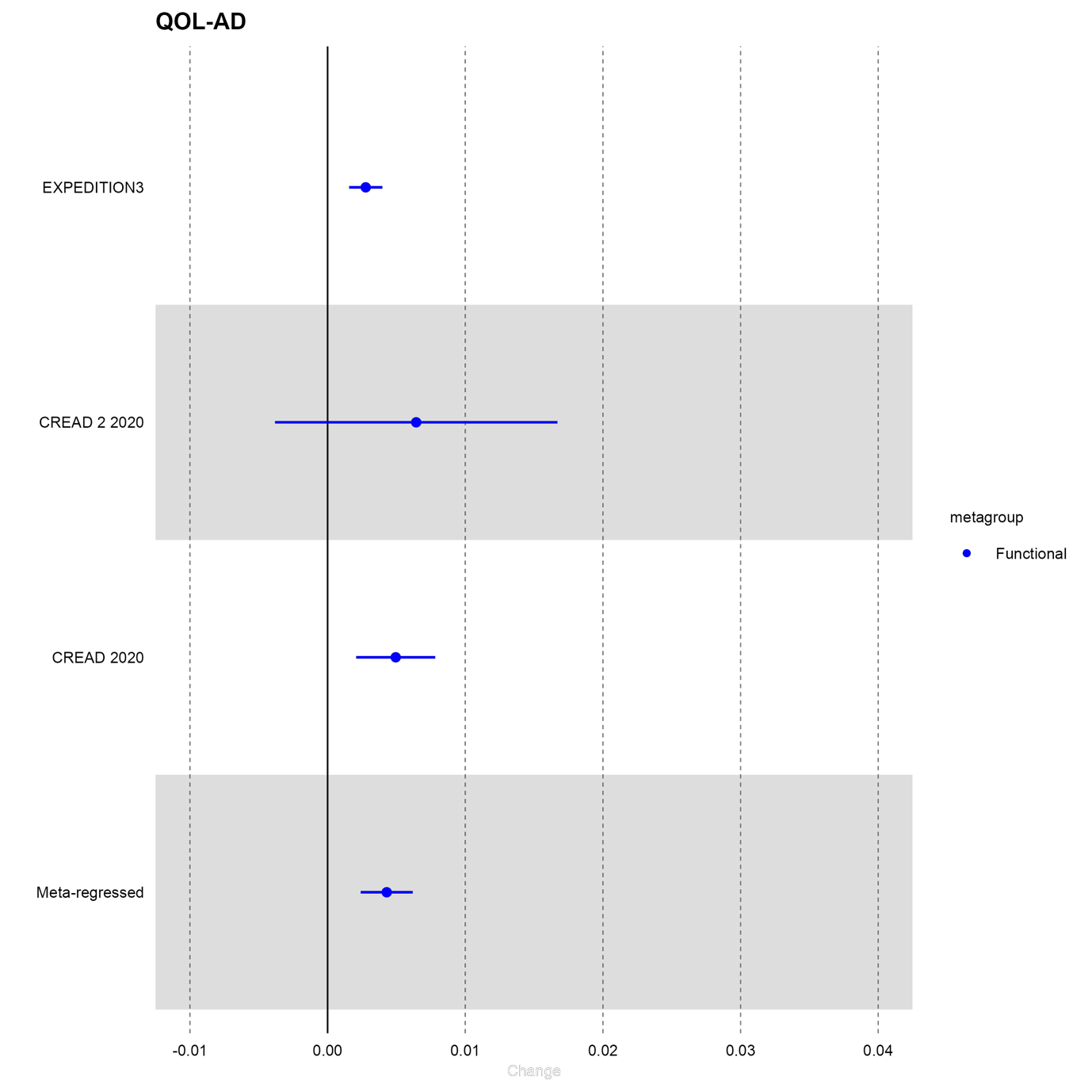


*FIGURE S8: Meta-analysis of ADAS-Cog-11 tool which comprises primarily Cognitive measures. N=4007 across 10 studies.*


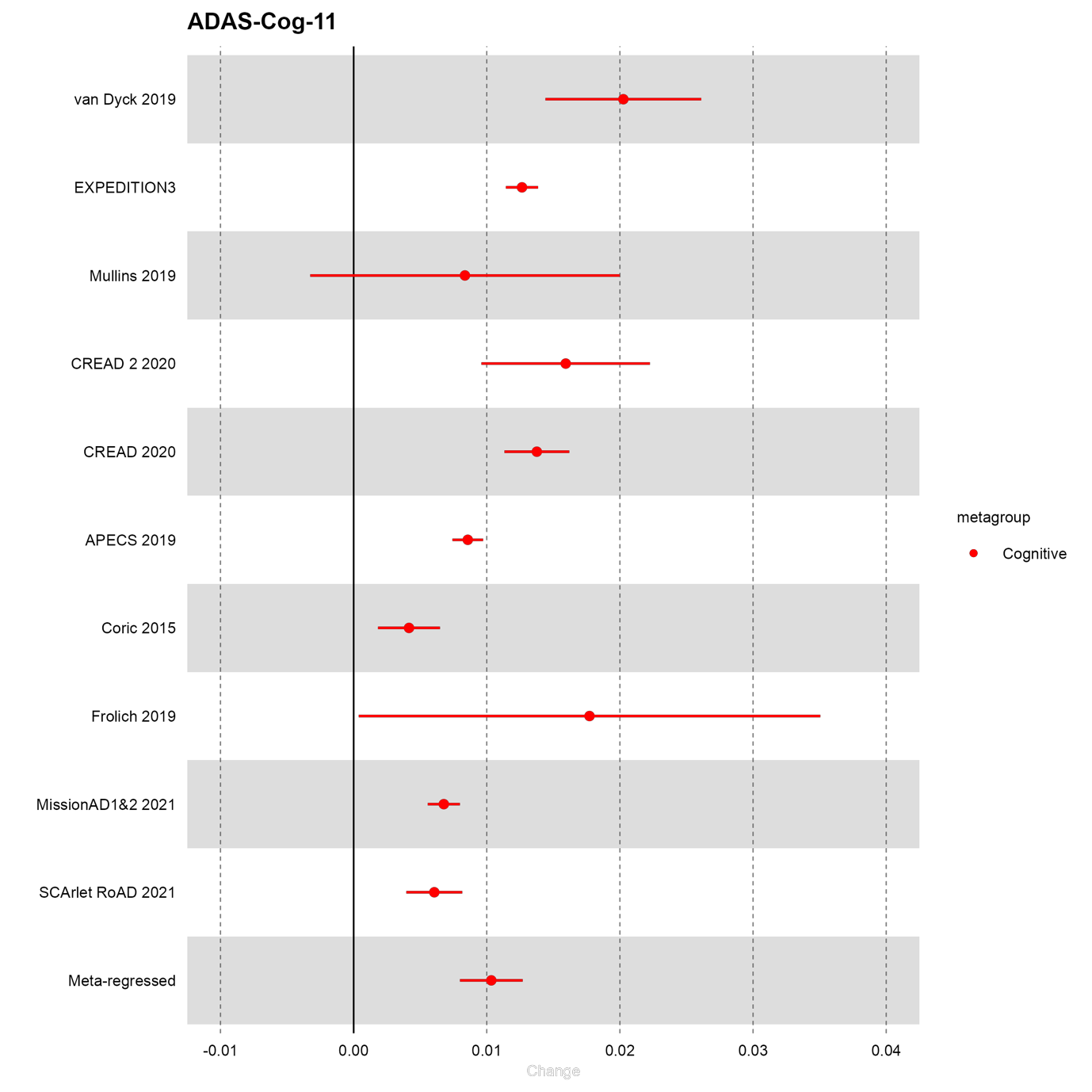


*FIGURE S9: Meta-analysis of ADAS-Cog-13 tool which comprises primarily Cognitive measures. N=4103 across 11 studies.*


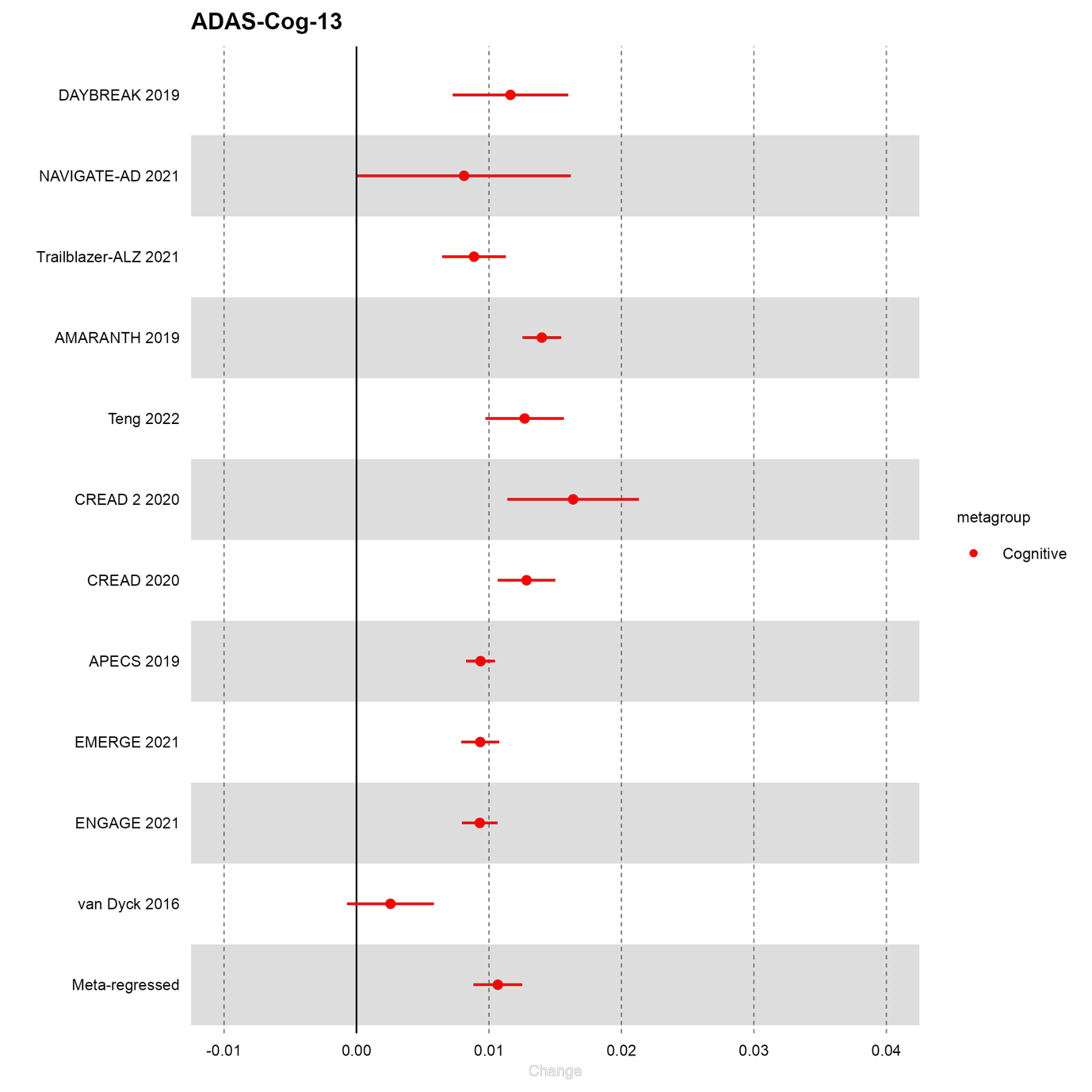


*FIGURE S10: Meta-analysis of ADAS-Cog-14 tool which comprises primarily Cognitive measures. N=2438across 4 studies.*


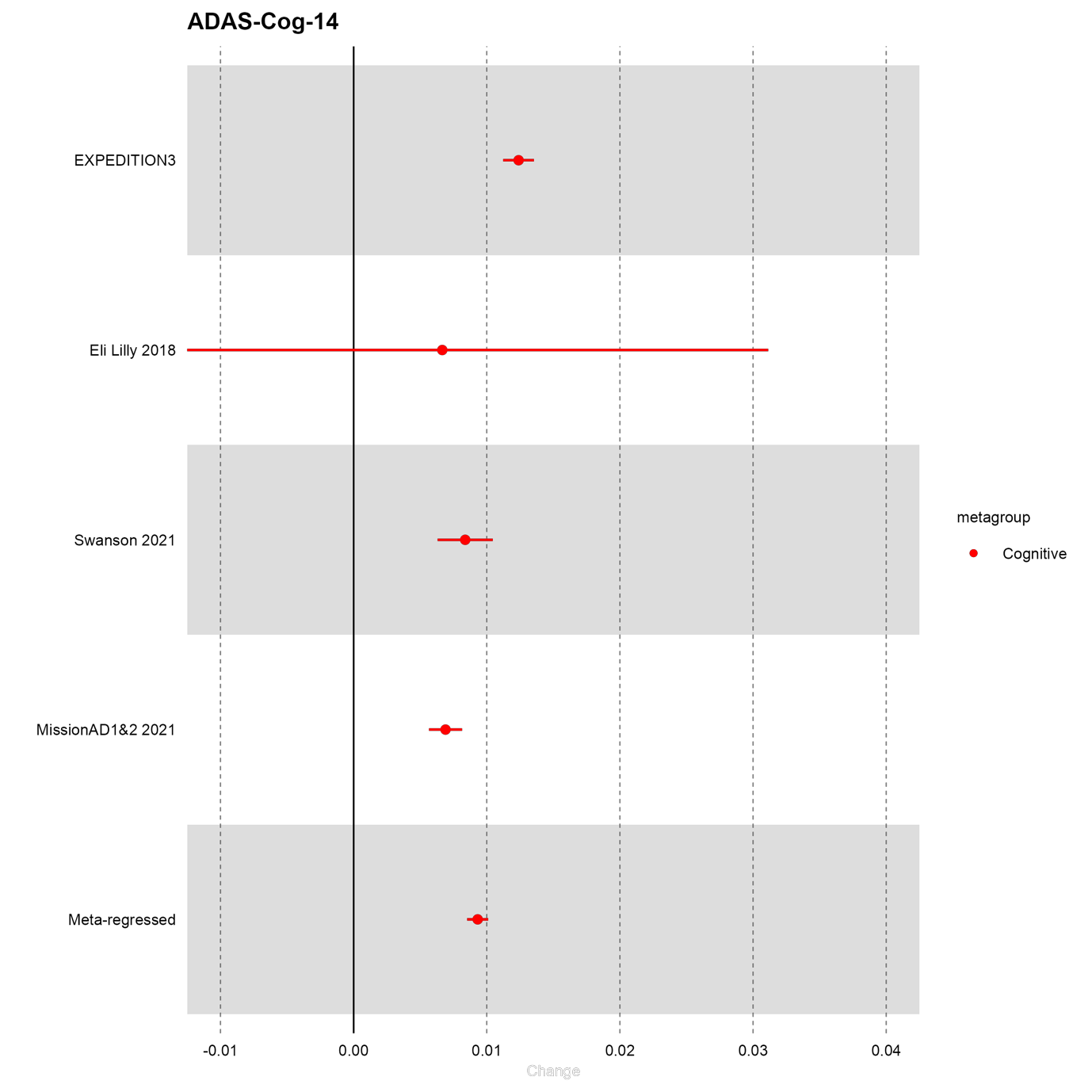


*FIGURE S11: Meta-analysis of MMSE tool which comprises primarily Cognitive measures. N=6343 across 16 studies.*


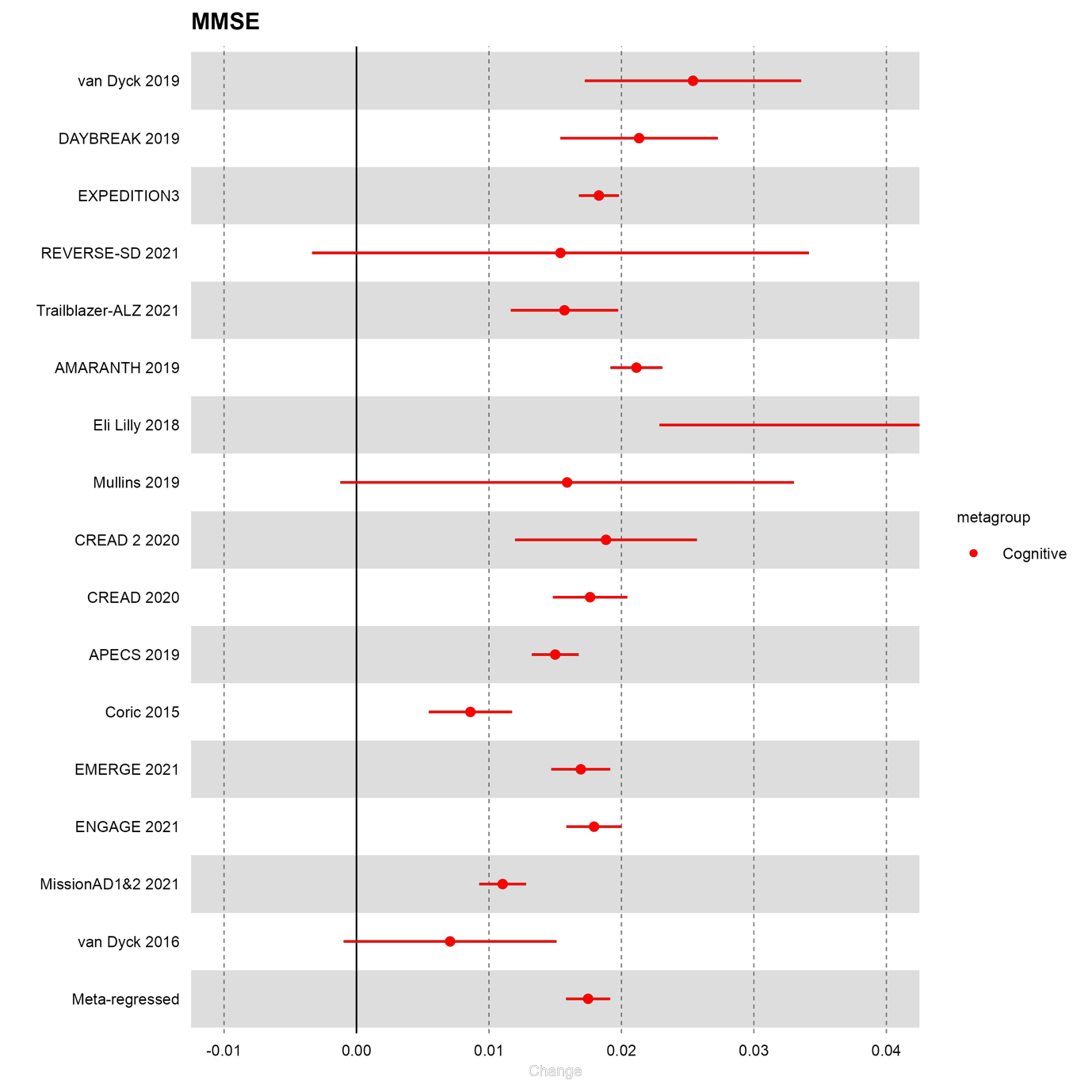


*FIGURE S12: Meta-analysis of CDR-SB tool which comprises Both cognitve and functional domains. N=7215 across 20 studies.*


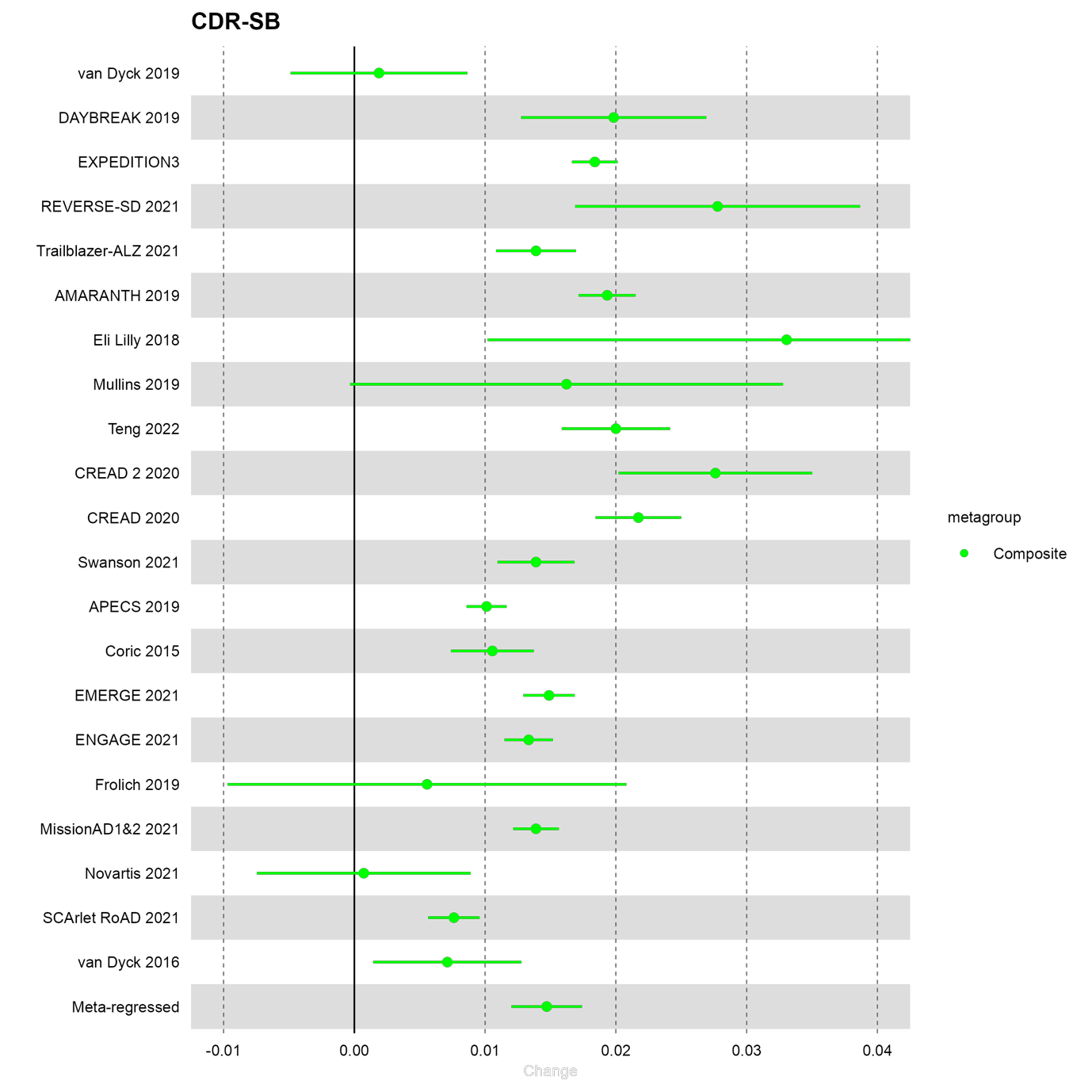


*FIGURE S13: Meta-analysis of iADRS tool which comprises Both cognitve and functional domains. N=2633 across 5 studies*


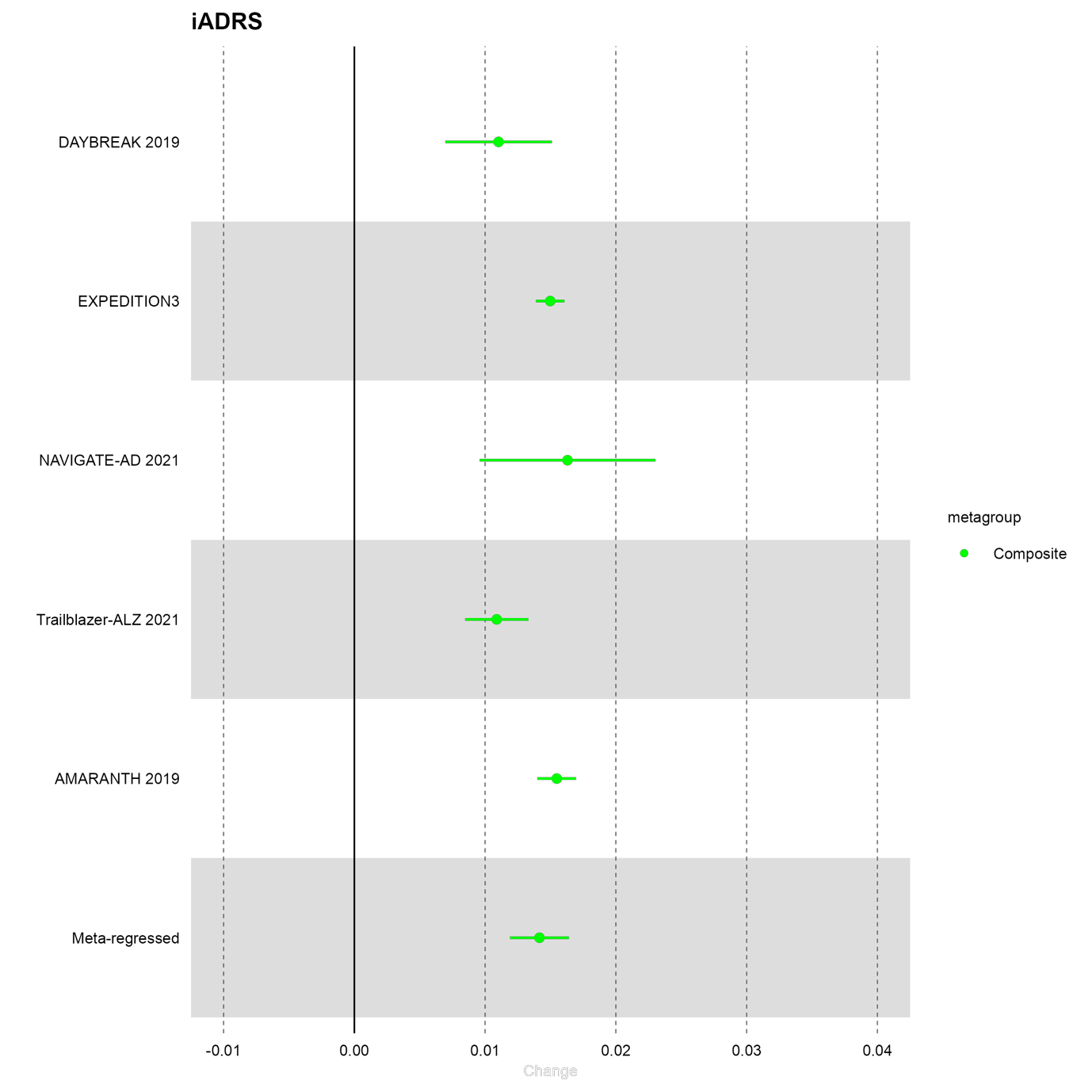


*FIGURE S14: Meta-analysis of NPI tool which primarily asseses neuropsychiatric domains. N=3226 across 7 studies*


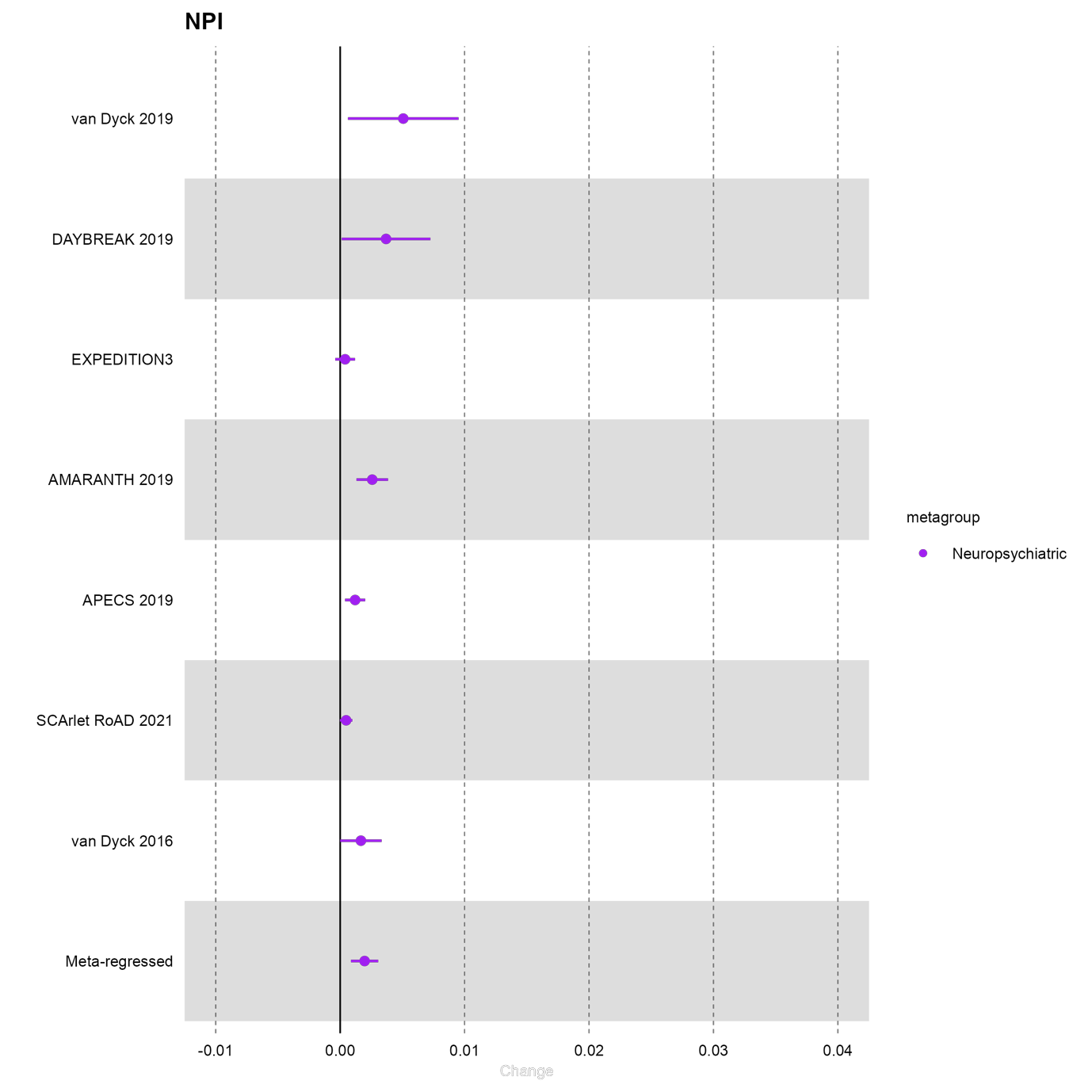


## **References**

1. McGhee DJM, Ritchie CW, Thompson PA, Wright DE, Zajicek JP, Counsell CE. A systematic review of biomarkers for disease progression in Alzheimer's disease. *PloS one* 2014; **9**(2): e88854.

2. Higgins JP, Thomas J, Chandler J, et al. Cochrane handbook for systematic reviews of interventions: John Wiley & Sons; 2019.

3. Sterne JA, Savović J, Page MJ, et al. RoB 2: a revised tool for assessing risk of bias in randomised trials. *bmj* 2019; **366**.

4. Cummings JL, Zhong K, Kinney JW, et al. Double-blind, placebo-controlled, proof-of-concept trial of bexarotene in moderate Alzheimer’s disease. *Alzheimer's Research & Therapy* 2016; **8**(1): 4.

5. van Dyck CH, Nygaard HB, Chen K, et al. Effect of AZD0530 on Cerebral Metabolic Decline in Alzheimer Disease: A Randomized Clinical Trial. *JAMA neurology* 2019; **76**(10): 1219-29.

6. Van Dyck C, Sadowsky C, Le Prince Leterme G, et al. Vanutide Cridificar (ACC-001) and QS-21 Adjuvant in Individuals with Early Alzheimer's Disease: Amyloid Imaging Positron Emission Tomography and Safety Results from a Phase 2 Study. *The Journal of Prevention of Alzheimer's Disease* 2016; **3**(2): 75-84.

7. Potter H, Woodcock JH, Boyd TD, et al. Safety and efficacy of sargramostim (GM‐CSF) in the treatment of Alzheimer's disease. *Alzheimer's & Dementia: Translational Research & Clinical Interventions* 2021; **7**(1): e12158.

8. Wang H-Y, Pei Z, Lee K-C, et al. Effects of simufilam on cerebrospinal fluid biomarkers in Alzheimer’s disease: A randomized clinical trial. 2021.

9. NCT02477800. 221AD301 Phase 3 Study of Aducanumab (BIIB037) in Early Alzheimer's Disease (ENGAGE). [*https://clinicaltrialsgov/ct2/show/study/NCT02477800*](https://clinicaltrialsgov/ct2/show/study/NCT02477800) 2015.

10. Sperling R, Henley D, Aisen PS, et al. Findings of Efficacy, Safety, and Biomarker Outcomes of Atabecestat in Preclinical Alzheimer Disease: A Truncated Randomized Phase 2b/3 Clinical Trial. *JAMA Neurology* 2021; **78**(3): 293-301.

11. Prins ND, Harrison JE, Chu H-M, et al. A phase 2 double-blind placebo-controlled 24-week treatment clinical study of the p38 alpha kinase inhibitor neflamapimod in mild Alzheimer’s disease. *Alzheimer's Research & Therapy* 2021; **13**(1): 106.

12. Frölich L, Wunderlich G, Thamer C, Roehrle M, Garcia M, Dubois B. Evaluation of the efficacy, safety and tolerability of orally administered BI 409306, a novel phosphodiesterase type 9 inhibitor, in two randomised controlled phase II studies in patients with prodromal and mild Alzheimer’s disease. *Alzheimer's Research & Therapy* 2019; **11**(1): 18.

13. Mullins RJ, Mustapic M, Chia CW, et al. A Pilot Study of Exenatide Actions in Alzheimer's Disease. *Curr Alzheimer Res* 2019; **16**(8): 741-52.
